# Smart Simulation Solutions for Hybrid Solar Engine Performance

**DOI:** 10.1021/acsomega.6c08338

**Published:** 2026-10-20

**Authors:** Miguel A. Rodriguez-Cano, Iñaki Requena-Leal, Justo Lobato, Manuel A. Rodrigo

**Affiliations:** Department of Chemical Engineering, Faculty of Chemical Sciences & Technologies, 16733 Universidad de Castilla-La Mancha, Ciudad Real 13071, Spain

## Abstract

This work presents the development and validation of a lightweight process simulator capable of reproducing the behavior of a solar-hybrid engine by integrating four computational modules: photovoltaic energy generation, electrochemical energy storage, engine energy supply, and engine operation. The models are solved using a first-order Euler discretization across multiple operating scenarios representative of real hybrid-engine control modes. Model parameters were obtained through time-series fitting of local environmental data, adjustment of solar-panel performance using manufacturer specifications, and targeted experiments isolating the behavior of each subsystem. The simulator accurately reproduced the performance of two hybrid-engine prototypes, an agricultural irrigation system, and an automotive test platform, with correlation coefficients of approximately 0.981 for energy-related variables and 0.926 for chemical species concentrations. The strong agreement between predicted and experimental results demonstrates the robustness of the modeling approach and supports its potential application as an energy-management tool for future solar-hybrid engine systems. To the best of our knowledge, this is the first model in which a chloralkaline process is integrated with solar photovoltaic panels for H_2_ production and applied in two different scenarios, with the addition of carbon capture within the same procedure.

## Graphical abstract

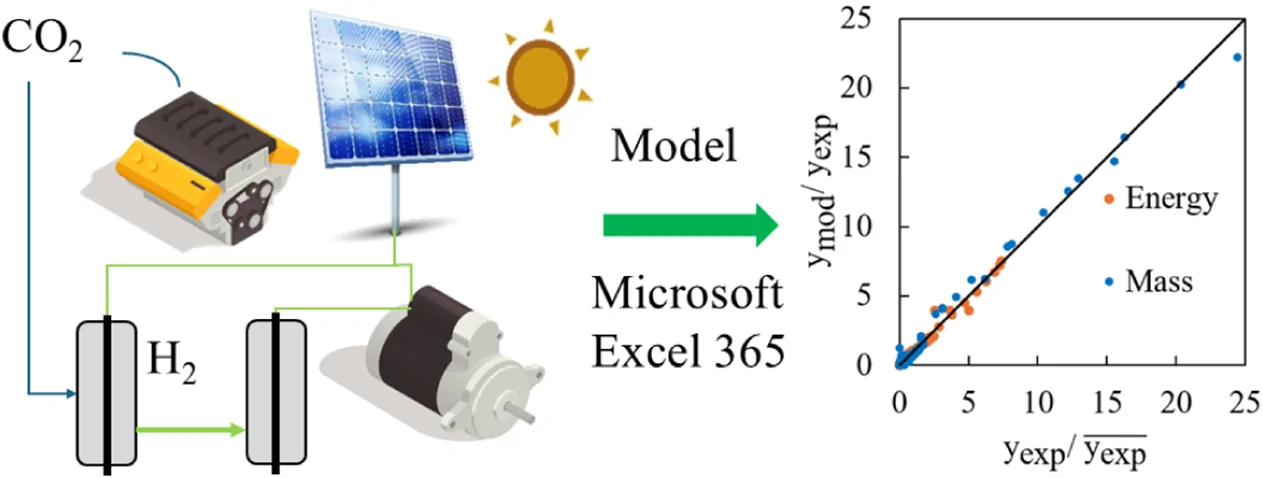

## 1 Introduction

For several decades, the use of process simulators in various chemical engineering applications has steadily increased.^1^ These simulators, which can incorporate phenomenological and/or empirical models, have become essential tools for the design and modification of process units and industrial plants, as well as for their operational control. In this context, their contribution to process optimization, not only from an economic perspective but also from an operational one, is emerging as a key driver of future innovation. Therefore, understanding the governing mechanisms of the main processes during the modeling stage, together with the development of robust and, whenever possible, simple mathematical models, is crucial to ensure their applicability. Likewise, the development of user-friendly simulation and optimization tools based on these models is of paramount importance.

Perhaps one of the areas currently attracting the greatest interest within chemical engineering is the development of energy management systems.^2^ This growing attention stems from the fact that energy has become a key variable in the transition toward a more sustainable society, where the efficient use of both material and energy resources is a primary objective.^3–5^ In particular, the management of energy derived from renewable and conventional sources is of increasing importance, as it may form the basis for the efficient operation of hybrid energy systems.^6^ This need has become especially evident as the development of such systems continues to accelerate and as the importance of an appropriate energy mix has gained widespread recognition. Rather than relying exclusively on a single energy source, a balanced combination of technologies is increasingly regarded as a more realistic and effective pathway toward sustainability. In this context, it is important to recognize that the intermittent nature of many renewable energy sources limits their suitability as stand-alone solutions. Consequently, the hybridization of energy systems emerges as one of the most feasible and promising strategies for the coming years.

The impact becomes even more significant when considering the optimized use of electricity from the grid. One of the main strategies employed for this purpose is the implementation of time-dependent electricity pricing schemes, which encourage the use of renewable and lower-cost energy sources while reducing reliance on generation technologies primarily dedicated to grid stabilization and demand balancing. In this context, local energy-storage systems play a crucial role. These systems are commonly based on electrochemical technologies and include various types of batteries (discontinuous electrochemical reactors), redox-flow batteries, and electrolyzer-fuel-cell systems, which can be regarded as the continuous-operation counterpart of conventional batteries.

The different characteristics of energy-storage technologies, particularly their widely varying capacities and dynamic response times, together with the strong temporal fluctuations in energy prices, have created a highly relevant field of research. The selection of an appropriate operating strategy can lead not only to significant cost savings but also to the development of more efficient and resilient energy systems. Among the available storage options, hydrogen produced by water electrolysis using photovoltaic solar energy has emerged as one of the most promising alternatives because of its high efficiency, long service life, and potential for large-scale energy storage.^7,8^

Recently, our research group has proposed two innovative applications that provide the main motivation for the work presented in this article. The first concerns the development of a low-TRL technology (TRL 3–4) based on hybrid solar power systems that integrate photovoltaic energy, saline electrolysis, and fuel cell technologies. When renewable energy is insufficient, the system can be supplemented by fossil-fuel-powered thermal engines and, where available, electricity from the grid. The primary objective of this concept is to maximize the utilization of solar energy while minimizing the overall carbon footprint. To achieve this, excess solar energy is used to produce hydrogen through electrolysis, generating a highly alkaline solution as a byproduct. When fossil fuels must be used to compensate for energy shortages, the carbon dioxide released during combustion is captured through reactive absorption in this alkaline solution. In this way, the system combines energy production, energy storage, and carbon capture within a single integrated framework.^9,10^

The technology has been successfully validated through case studies in the automotive and agricultural irrigation sectors, demonstrating its considerable potential. First, its application in the automotive industry^11^ suggests that, with further technological development and a significant increase in technology readiness level (TRL), it could provide an alternative to conventional battery-based systems and/or PEM electrolyzer-fuel-cell configurations commonly used in urban mobility. In such applications, vehicles could continuously harvest and store energy whenever exposed to sunlight, whether in operation, parked, or idle. Second, its implementation in irrigation pumping systems^12^ offers a complementary solution to pumps powered by diesel engines or diesel generator sets. In this case, solar energy collected during daylight hours can be stored in the electrochemical device and later utilized at night, when irrigation is generally more efficient due to the substantially lower water losses associated with evaporation under high daytime temperatures. In this scenario, any additional energy demand could be supplied by fossil fuel, with a reduction in CO_2_ emissions as a consequence of its transformation into carbonates/bicarbonates.

In both case studies, the need to integrate solar radiation forecasting tools with simulation models of the different components of the hybrid engine has become evident. These tools can subsequently be coupled with an optimization framework capable of identifying and recommending the most appropriate operational actions. This work describes the key aspects involved in the development of the simulation tools and their calibration and adaptation to accurately reproduce the performance observed in experimental case studies.

Although the development of each individual element has been addressed in previous studies, the integration and, above all, the performance assessment reveal the need for further applications to be developed and highlight the enormous impact they may generate in the energy transition, which has now become one of the main transformative drivers of society.

To the best of our knowledge, the model developed in this work for the two case studies represents the first mathematical framework integrating weather forecasting with solar photovoltaic power generation. The electricity produced can be either used directly or supplied to an EDEN chlor-alkali electrolyzer for the production of H_2_, Cl_2_, and OH^–^. The generated H_2_ is stored and subsequently used to supply a fuel cell, while a combustion engine covers potential energy deficits. An additional advantage of the proposed system is the capture of CO_2_ through the utilization of the OH^–^ produced during the electrolysis process.

## 2 Experimental Section

### 2.1 Solar Hybrid Engine Process Description

The hybrid solar engine system consists of several interconnected devices, all of which are illustrated in Figure 1. The primary components include the solar photovoltaic panels, the EDEN saline PEM electrolyzer, a PEM fuel cell stack, the electric motor, the combustion engine, storage tanks, gasometers, compressors and pressurized cylinders, and the associated transmission system. Detailed performance data for these units, as well as their operational integration, are provided elsewhere for applications in both automotive propulsion and agricultural irrigation.^11,12^ The operating conditions of the electrochemical devices are described below. Solar PV panels directly supply energy to the electric motor, and any surplus electricity is directed to the EDEN electrolyzer. The electrolyzer is operated using a 2 M NaCl solution as the electrolyte in both the anodic and cathodic compartments. In the cathode compartment, the expected reaction is water reduction (eq R1), producing hydrogen (H_2_) via the hydrogen evolution reaction (HER). Meanwhile, in the anode compartment, chloride ions are oxidized to chlorine (eq R2). The chlorine generated subsequently reacts with hydroxide ions according to the disproportionation shown in eq R3 to form chloride ions and hypochlorous acid. In addition, water oxidation also takes place at the anode, competing for electrons through the oxygen evolution reaction (OER) (eq R4). The hydrogen produced is compressed and stored in a dedicated cylinder. The alkaline solution enriched in OH^–^ from the cathodic water reduction is also collected for later use in carbon dioxide capture via a reactive absorption process. When solar energy is insufficient to meet the electric motor demand, the PEM fuel cell stack supplies the required power by consuming the stored hydrogen and atmospheric air. Once hydrogen storage is depleted, an integrated combustion engine provides energy to the electric drivetrain through the appropriate transmission system. The combustion engine emits CO_2_, which is directed to the alkaline reservoir (eq R5). In this stage, the reservoir acts as a reactive absorption system, capturing CO_2_ with the accumulated OH^–^ ions and forming sodium carbonate or sodium hydrogen carbonate, depending on the CO_2_ to OH^–^ ratio.

**Figure 1:**
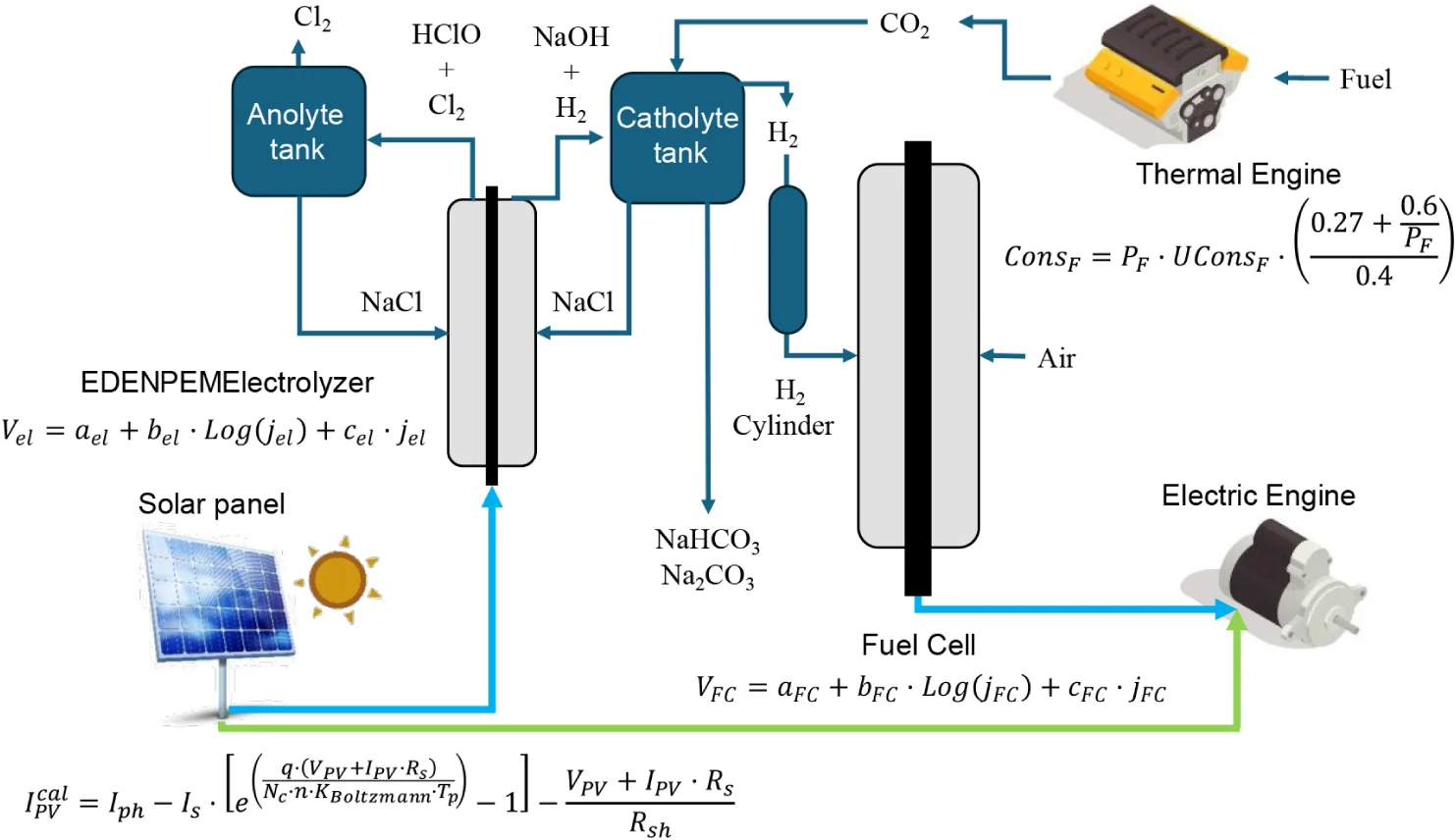
Scheme of the installation with the main components: photovoltaic solar panel, EDEN PEM electrolyzer with the anolyte and catholyte (NaCl 2 M) tanks, PEM Fuel Cell, thermal and electric engine which cover the consumption.

R1 $$H_{2}O+e^{-}\to 1/2H_{2}+OH^{-}$$

R2 $$2Cl^{-}\to Cl_{2}+2e^{-}$$

R3 $$Cl_{2}+OH^{-}\to Cl^{-}+HClO$$

R4 $$H_{2}O\to 1/2O_{2}+2H^{+}+2e^{-}$$

R5 $$CO_{2}+xNaOH\to Na_{2}CO_{3}/NaHCO_{3}$$

The electrolyzer operated continuously except during periods with no solar irradiation or when all available photovoltaic power was required to supply the electric motor. Based on this premise, five distinct operating scenarios were defined for the system:

1. No energy generation or consumption: Neither solar power production nor engine operation takes place.
2. Electric motor powered by solar energy with surplus to the electrolyzer: The electric motor runs exclusively on solar power, and any excess electricity is directed to the electrolyzer.
3. All solar power dedicated to the electric motor: The motor uses the full photovoltaic output, leaving no surplus energy for hydrogen production.
4. Electric motor powered by solar energy with supplemental fuel cell support: The fuel cell provides additional power when solar generation is insufficient to meet the motor demand.
5. Combustion engine operation: Activated when neither solar energy nor stored hydrogen is available to supply the electric motor.
6. Grid-connected hydrogen production (applied only in the second case study): The system is connected to the electrical grid to operate the electrolyzer for hydrogen generation.

A similar system with renewable energy generation, energy storage (in a battery and H_2_) have been developed and studied to feed H_2_ and electricity to a methanol production plant.^13^

### 2.2 Mathematical Model Description (Model Formulation)

The model implemented in the simulator of the solar hybrid engine energy-management system is, in turn, composed of several submodels that allow the representation of all relevant processes:

1. a solar photovoltaic energy-generation model;
2. a saline electrolyzer model;
3. a fuel-cell stack model;
4. a combustion-engine and reactive-absorption system model.

### 2.3 Photovoltaic Energy Generation Model

This model, in turn, consists of two submodels: a predictive model for solar irradiance and temperature (a climate predictor) based on time-series analysis, and a second model that, using data obtained from solar panels of different capacities, is able to predict the electrical power supplied by the panels to both the electric motor and the electrolyzer.

Solar panels served as the main power source of the system. The parameters used in the simulation correspond to real operating data obtained from different photovoltaic modules, as summarized in Table S1. Solar irradiance is a key variable in the model; therefore, a realistic approach based on the sunrise and sunset times at the winter and summer solstices, together with the day of the year, was employed.^14^ The solar irradiance, λ, was estimated using eq 6, which incorporates the time of day *t* expressed in hours (from 0.00 at 00:00 h to 23.99 at 23:59 h), the day of the year *n*_*day*_ (ranging from 0 to 365), the maximum and minimum annual irradiance values λ_max_ and λ_min_, set to 1500 and 500 W·m^–2^, respectively, and an empirical fitting parameter φ_3_, set to −65. The sunrise and sunset times, *t*_*sunrise*_ and *t*_*sunset*_, respectively, were calculated using eq S1 and eq S2. These expressions were derived from reference values corresponding to sunrise and sunset during the winter and summer solstices. The ambient temperature profile is also an important parameter, as both efficiency and operating pressure (of the hydrogen storage) depend on it. The temperature was calculated using eq 7, where *T*_*min*_ and *T*_*max*_ correspond to the minimum and maximum temperatures (in K) for the selected day, *n*_*day*_. The temperature at the surface of the photovoltaic panel was also estimated, since the electrical power generated by the panel varies significantly with its operating temperature. The same model used for the ambient temperature was applied to the panel surface temperature, and it was evaluated using eq S3. This model was validated in previous work by our group.^15^

1 $$\lambda \left(W\;m^{-2}\right)=\sin\left(\left(\frac{\pi }{\left(t_{sunset}-t_{sunrise}\right)}\right)\cdot \left(\frac{t}{3600}-t_{sunrise}\right)\right)\cdot \left(\lambda_{min}+\left(\lambda_{max}-\lambda_{min}\right)\cdot \frac{1}{2}\cdot \left(1+\sin\left(\frac{2\cdot \pi \cdot \left(n_{day}+\varphi_{3}\right)}{365}\right)\right)\right)\cdot \left(1-Cloud\cdot Rnd\left(1\right)\right)$$

2 $$T\left(K\right)=T_{min}+\left(T_{max}-T_{min}\right)\cdot \frac{1}{2}\cdot \left(1+\sin\left(\frac{2\cdot \pi \cdot \left(n_{day}+\varphi_{3}\right)}{365}\right)\right)\cdot \left(\sin\left(\frac{0.7\cdot \pi }{t_{sunset}-t_{sunrise}}\right)\cdot \left(\frac{t}{3600}-t_{sunset}-4\right)\right)$$

Once the solar irradiance and the surface temperature of the PV panels were determined, the electrical power generated by the modules was calculated. The energy-estimation model is based on the equivalent circuit of a photovoltaic panel, shown in Figure S1, and the characteristic I–V relationship described by eq 8. In this equation, *V*_*PV*_ and *I*_*PV*_ represent the output voltage and current of the photovoltaic module (parameters in Table S1); *q* is the elementary charge; *N*_*c*_ the number of cells; *n* the diode ideality factor (Table S1); *k*_*Boltzmann*_ the Boltzmann constant; and *T*_*p*_ the panel surface temperature obtained from eq S3. The series and shunt resistances are denoted as *R*_*s*_ and *R*_*sh*_, respectively (see Figure S1). The photocurrent *I*_*ph*_ was calculated using eq S4, while the reverse saturation current *I*_*s*_ was obtained from eq S5, and the cell saturation current *I*_*rs*_ from eq S6. To solve eq 8, an iterative approach was employed in which *I*_*PV*_ was replaced by $I_{PV}^{cal}$ until the difference between consecutive values was smaller than 0.01. A maximum of 500 iterations was allowed, and any negative current value obtained during the computation was set to zero. The power delivered by a single photovoltaic panel was obtained by multiplying its output current and voltage, as expressed in eq S7. For configurations where panels were connected in series or in parallel, the output voltage or current, respectively, was scaled by the number of panels according to eq S8 and eq S9. The total power generated by the complete PV array was then calculated using eq S10. An energy balance was applied to determine the number of photovoltaic panels available for hydrogen production when the electric motor was operating simultaneously. This value was computed by subtracting the power consumption of the water pump, *P*_*cons*_, from the total power produced by the PV array (eq S10) and dividing the result by the power output of a single panel (eq S7), as shown in eq 9. The actual power supplied to the electrolyzer was calculated by multiplying the integer part of the number of available panels, denoted by the superscript “^int^”, by the power of a single PV panel (eq S7), as expressed in eq 10. If the difference between the generated power and the consumption was zero or negative, the number of PV panels connected to the electrolyzer was set to zero. This model is similar to that used by Saadaoui et al. 2021.^16^

3 $$I_{PV}^{cal}=I_{ph}-I_{s}\cdot \left[e^{\left(\frac{q\cdot \left(V_{PV}+I_{PV}\cdot R_{s}\right)}{N_{c}\cdot n\cdot K_{\mathrm{Boltzmann}}\cdot T_{p}}\right)}-1\right]-\frac{V_{PV}+I_{PV}\cdot R_{s}}{R_{sh}}$$

4 $$N_{PV}^{H}=\frac{P_{PV}^{matrix}-P_{cons}}{P_{PV}}$$

5 $$P_{H}=P_{PV}\cdot N_{PV}^{H,int}$$

### 2.4 Electrochemical Energy Storage Model

The second model corresponds to the electrolyzer, responsible for storing energy in the form of hydrogen and for producing chlorine and an alkaline solution. The electrolyzer module operates using the power supplied by the photovoltaic panels connected to it (eq 10). The cell voltage, *V*_*el*_, was calculated using eq 11, where *a*_*el*_, *b*_*el*_, and *c*_*el*_ are experimentally determined model parameters, and *j*_*el*_ is the current density, initially estimated using eq S11. The difference between the estimated current density and the value derived from the calculated power and voltage was then evaluated using eq S12, and the model iteratively minimized this difference to below 0.0001, with a maximum of 500 iterations. Once convergence was achieved, the electrolyzer current was obtained from eq S13, and the corresponding power was computed using eq S14.

6 $$V_{el}=a_{el}+b_{el}\cdot \log\left(j_{el}\right)+c_{el}\cdot j_{el}$$

### 2.5 Engine Powering Model

The fuel cell operated using the hydrogen accumulated by the electrolyzer whenever the solar power available was lower than the engine demand or during periods without solar irradiation. In this case, the voltage and current of the fuel cell were calculated using an approach analogous to that applied to the electrolyzer. First, the required electrical power was obtained by subtracting the instantaneous solar power from the total power demand (eq S15). The fuel-cell voltage, *V*_*FC*_, was then computed using eq S16, an expression similar to the electrolyzer voltage equation (eq 11). The empirical parameters *a*_*FC*_, *b*_*FC*_, and *c*_*FC*_ were determined experimentally, and *j*_*FC*_ represented the current density of the fuel cell, initialized at 30 mA cm^–2^. A similar iterative estimation procedure (eq S17) was subsequently applied to obtain the operating current and power of the fuel cell, *I*_*FC*_ and *P*_*FC*_, calculated using eq S19 and eq S20, respectively. When the total energy demand exceeded both the stored hydrogen capacity and the solar power generation, the required energy was supplied by a combustion engine. The total power that this device had to deliver, *P*_*F*_, was calculated using eq S21 as the difference between the power demand of the water pump, *P*_*cons*_, and the power generated by the photovoltaic array, $P_{PV}^{matrix}$ , which could be zero when the system operated after sunset. The corresponding fuel flow rate was then estimated using eq 12, where *Cons*_*F*_ denotes the hourly fuel consumption and *UCons*_*F*_ represents the specific fuel consumption required to generate one watt of power.

7 $$Cons_{F}=P_{F}\cdot UCons_{F}\cdot \left(\frac{0.27+\frac{0.6}{P_{F}}}{0.4}\right)$$

### 2.6 Engine Performance Model

The accumulated energy for all subsystems was quantified using a unified model expression, given in eq 13. In this formulation, $E_{x}^{t}$ represents the energy of device *x* at time *t*, where *x* may correspond to the photovoltaic array (eq S22), the electrolyzer (eq S23), the fuel cell (eq S24), the combustion engine (eq S25), or the total energy consumed (eq S26). The term $E_{x}^{t-\Delta t}$ denotes the corresponding energy at the previous time step, *t* – Δ*t*. The instantaneous power of each subsystem, *P*_*x*_, was incorporated through the specific model equations: eq S10 for photovoltaic power, eq S14 for the power consumed by the electrolyzer, eq S20 for the power delivered by the fuel cell, and eq S21 for the power generated by the combustion engine. Mass balances for all reactants and products were defined according to eqs S27–S40. The hydrogen inventory at time *t*, $H_{2}^{t}$ , was calculated using eq S27, accounting for the stored hydrogen, its production in the electrolyzer, and its consumption in the fuel cell, each corrected with the respective device efficiencies; input and output flow rates were included in the formulation but assumed to be zero for this case study. For chlorine, the concentration in the electrolyte, [Cl_2_]^*t*^, was determined using eq S28 under the assumption that all generated chlorine gas remained dissolved in the liquid phase and that no disproportionation of this compound occurred, i.e., eq R2 took place whereas eq R3 did not. This simplification was adopted because chlorine was not utilized, and its chemical state was therefore irrelevant to the analysis. Unlike hydrogen, chlorine was not consumed at the fuel cell cathode because atmospheric air served as the oxidant; consequently, its concentration increased monotonically over time. The hydroxide ion concentration was evaluated similarly using eq S29, while the total amount of hydroxide (in moles) was obtained using eq S30. The total fuel consumption of the combustion engine was computed using eq S31, and the corresponding carbon dioxide generation was estimated with eq S32; the accumulated CO_2_ emissions were then obtained using eq S33. Finally, regarding the capture of CO_2_ by the hydroxide ions produced in the electrolyzer, two scenarios were considered. When CO_2_ was present in excess, its concentration after capture was calculated using eq S34. Under these conditions, all hydroxide ions were consumed, resulting in a final hydroxide concentration of zero, while the amount of carbonate/hydrogen carbonate formed was determined using eq S35. Conversely, when CO_2_ was the limiting reagent, its concentration after capture was zero, and the remaining hydroxide concentration was calculated using eq S36. The corresponding amount of carbonate/hydrogen carbonate generated was then estimated using eq S37.

8 $$E_{x}^{t}=E_{x}^{t-\Delta t}+P_{x}\cdot \frac{\Delta t}{3600}$$

### 2.7 Error of the Model and Sensitivity Analysis

The goodness of the model predictions was evaluated using several statistical metrics. First, the coefficient of determination, R^2^, (eqs S38–S40) was calculated for all the model output data, considering both matter and energy balances. In addition, the root-mean-square error, RMSE, (eq S41) and the mean absolute error, MAE, (eq S42) were calculated and analyzed for the different simulated parameters individually: H_2_ pressure, Cl_2_ concentration, OH^–^ production, fuel consumption, CO_2_ generation, and carbonate/bicarbonate formation for the matter balance. For the energy balance, the parameters corresponding to the electrolyzer, fuel cell, total energy consumption, and thermal engine were considered.

A sensitivity analysis was conducted by varying the Δt parameter (30, 150, 300, 600, 1200, and 2400 s) and estimating the root-mean-square relative error, RMSRE (eq S43) of the predicted values relative to those obtained using the smallest time step (30 s).

### 2.8 Modeling of the Operating Conditions

Table 1 presents the main characteristics of the different hybrid engine operating scenarios and the modeling approach applied to one of them.

**Table 1:** Operating Scenarios of the Solar Hybrid Engine

Scenario definition	Modeling
0-Stand-by	All the systems are idle
1-The number of solar panels supplied sufficient energy to drive the electric motor, while at least one panel was available for hydrogen production; the fuel cell remained inactive	The total solar power was calculated using eq S7 (based on the results of eq 8 and eqs S4–S6). The power required by the electric water pump was then subtracted, and the number of panels allocated to hydrogen production was determined using eq 9 and eq 10. Subsequently, the electrolyzer current, voltage, and power were evaluated using eq 11 and eqs S11–S14. In this scenario, the fuel cell and combustion engine consumption were zero (I_FC_ = 0, V_FC_ = 0, Cons_F_ = 0). The corresponding energy balances are presented in eq S22, eq S23, and eq S26, where Δt represents the simulation time step. Mass balances were obtained using eq S27, eq S30, and eqs S34–S37
2-All power generated by the solar panels was used exclusively to supply the electric motor	The electrochemical devices (electrolyzer and fuel cell) and the combustion engine were not operating (I_el_ = 0, V_el_ = 0, I_FC_ = 0, V_FC_ = 0, Cons_F_ = 0). The photovoltaic panel current, voltage, and power were calculated using eq 8 and eqs S4–S7. The energy balances were obtained from eq S22 and eq S26. In this case, no products were generated or consumed
3-When the photovoltaic system produced power insufficient to meet the total system demand, but hydrogen was available in storage, the fuel cell supplied the remaining required power	Under these conditions, the electrolyzer and combustion engine remained inactive (I_el_ = 0, V_el_ = 0, Cons_F_ = 0). Photovoltaic panel current, voltage, and power were determined using eq 8 and eqs S4–S7, while fuel cell performance was evaluated using eqs S15–S20. The corresponding energy balances were computed using eq S22, eq S24, and eq S26, and the mass balances using eq S27 and eqs S34–S37
4-If no sunlight was available (or if solar power output fell below the required threshold) and stored H_2_ was insufficient, the combustion engine was used as the sole power source, while all other electric devices remained inactive (I_PV_ = 0, V_PV_ = 0, I_el_ = 0, V_el_ = 0)	When photovoltaic panels were producing power, their operating conditions were calculated using eq 8 and eqs S4–S7, while combustion engine performance was obtained using eq 13 and eq S21. Energy balances were computed with eq S22, eq S25, and eq S26, and mass balances using eqs S31–S37
5-Electrolyzer connected to the grid and rest of systems remais idle (only for Case study 2, hybrid vehicule)	When the electrolyzer is connected to the grid to produce H_2_for the car, only the electrolyzer operates; the rest of the system remains idle (I_PV_ = 0, V_PV_ = 0, I_FC_ = 0, V_FC_ = 0, Cons_F_ = 0). The electrolyzer conditions were estimated using eq 11 and eqs S11–S14 and the energy and mass balances were obtained from eqs S27–S30 and eq S23, respectively

### 2.9 Process Simulator and Model Parameter Estimation

The previous models have been implemented in Excel spreadsheets within a simulator programmed in Visual Basic for Applications (VBA). To do so, the differential equations resulting from the extensive-property balance were discretized using a first-order Euler method (conventional finite-difference discretization), with a time step of 600 s, which provided sufficient numerical stability for solving the system. At each time step, the system evaluates the total amount of energy received from sunlight and, based on this value, computes the energy produced by each solar panel using the energy-supply model for the motor. Then, depending on the scenario described in Table 1 in which the system is operating, it sends this information to the motor performance model and/or to the electrochemical energy-storage model, or alternatively connects both models. Figure S2 shows several illustrative screens of the software developed. To define the model parameters, the simulator was used by decoupling the nonrequired models, so that they would not interfere in the initial calibration:

1. The time-series model was calibrated using the geographical coordinates of the site where the case studies were conducted. The calibration was based on one year of meteorological data collected by an EasyWeatherPro weather station installed near the Enrique Costa Building (38° 59′ 43.01″ N, 3° 55′ 16.4″ W), together with data provided by the Spanish State Meteorological Agency (AEMET). The meteorological model was previously calibrated and validated in a recent work of the group.^15^
2. The parameters of the solar-panel models were fitted using the I–V curves of the different commercial panels employed, covering the power range from 10 to 400 W. These curves were used to characterize their electrical behavior under varying irradiance and temperature conditions. The parameters for this model were also previously obtained by our group.^15^
3. The parameters of the electrochemical energy-storage models were determined from targeted experiments performed on the electrolyzer, designed to capture its operational behavior under controlled conditions.
4. Finally, the parameters of the motor-performance model, whether the system was powered by hydrogen (fuel cell) or by nitromethane (combustion engine), were obtained by fitting experimental data from dedicated tests specifically carried out to isolate and characterize these effects.

The validation of the process simulator and the models implemented within it was carried out by assessing the agreement between the model predictions and the experimental data reported in two previously published case studies involving solar-hybrid engines: agricultural irrigation pumps and automotive applications.

In the first case study, the simulator was used to reproduce the behavior of an irrigation pump that stores energy during daylight hours and operates overnight to increase irrigation efficiency by reducing water losses due to evaporation during the hottest part of the day. Because the pump operates at night, the solar panels cannot power it directly, and therefore Scenarios 2, 3, and 4 were applied. The solar panel used in this case was a flexible 100 W, 12 V module from Aysolar. The electrolyzer was a custom-designed three-compartment prototype stack with three cells, an integrated gasometer, and an electrode area of 36 cm^2^. The fuel cell was a commercial Horizon H-12 unit with 13 cells, a nominal output of 12 W (−7.8 V, 1.5 A), and an optimal hydrogen flow rate of 180 mL min^–1^. The electric drive consisted of a peristaltic pump powered by a 12 V, 80 W (10,000 rpm) electric motor, while the internal combustion engine was taken from a nitromethane-powered RC vehicle (Bycmo, Model X, China) with a cylinder volume of 2.5 cm^3^. The experiment was carried out on 27 May 2025, beginning at 06:00 and running for 25 h. During daylight, the solar panel powered the electrolyzer, generating hydrogen that was stored in 1-L cylinders up to a maximum pressure of 4 bar, using as many cylinders as required; chlorine (not used) and hydroxyl species were also produced. At night, irrigation took place from 02:00 to 06:30, during which the fuel cell operated with a hydrogen flow of 135 mL min^–1^ (close to the optimal value) to power the pump. When the stored hydrogen was depleted, the combustion engine took over to supply the required power. The CO_2_ generated by the combustion engine was bubbled through the hydroxyl solution for capture and conversion into carbonate/hydrogen carbonate. In this sense the system is designed to work with green energy, but it also acts as a carbon capture system when needed.^17^ Further details regarding the construction, configuration, and operation of this model irrigation pump prototype can be found elsewhere in the literature.^12^

In the second case study, three 10 W solar panels (Oumefar, China) were mounted on a model-car prototype. The electric motor, sourced from Shenzhen Nuochen Store (China), delivered 80 W at 10,000 rpm and 12 V. The fuel-cell stack used in this prototype was identical to the one employed in the irrigation-pump case. The internal combustion engine corresponded to that of an RC car (BYCMO, 1/10 scale, 4WD, aluminum chassis) powered by nitromethane (2.3 cm^3^ nitro engine). Its estimated mechanical output ranged from 500 to 900 W, with operating speeds between 20,000 and 38,000 rpm and an idle speed of approximately 5,000 rpm. Fuel consumption varied between 6 and 10 mL min^–1^ of nitromethane (16 wt % in methanol). Further details regarding the construction, configuration, and operation of this model-car prototype can be found elsewhere in the literature.^11^

The meteorological and solar photovoltaic models were previously validated.^15^ The data used for the calibration and validation of the electrolyzer and fuel cell models were extracted from the aforementioned studies, in which Case Study 1 ^12^ and Case Study 2 ^11^ were developed and tested. These studies provided individual experiments used for parameter estimation and model calibration, as well as the remaining data sets employed during the calibration process. For matter and chemical species balance calculations, Faraday’s law was applied as described in the model formulation.

If this model were applied to different scenarios, the main difference would be the geographical location, as this parameter determines the minimum and maximum temperatures, sunrise and sunset times, and solar irradiance levels. Furthermore, the characteristics of the equipment should also be taken into account, such as the electrode active area, electrolyte volume, fuel cell efficiency, and other system-specific parameters.

## 3 Results and Discussion

### 3.1 Case Study 1

The first case study involves an agricultural irrigation system. The first module of the simulator predicts solar radiation, ambient air temperature, and the surface temperature of the solar panel. These predictions are shown in Figure 2a for May 27, 2025. The temperatures were computed using eqs 7 and S3, with the model estimating a maximum air temperature of 35 °C at 17:30 and a maximum panel-surface temperature of 57 °C at 18:30, 1 h later (Figure 2a). For air temperature, the high accuracy of the model prediction can be clearly observed in Figure 2a; no experimental surface-temperature data were available for the panel. Solar irradiance is shown in Figure 2b. According to the model, irradiance increases from sunrise at 07:00 (eq S1) to a maximum at 14:30 (800 W·m^–2^) and then decreases until sunset at 21:30 (eq S2). This predicted profile closely matches the real irradiance data for May 27, with only minor deviations in the afternoon period, might be associated with a small shadow in the solar panels (Figure 2b). The next step is the estimation of the photovoltaic power generated using the solar-irradiance profile (Figure 2a) for a 100 W panel, applying eqs 8–10 and eqs S4–S6. The resulting power curve is shown in Figure 3. As expected, the maximum electrical output of the solar panel coincides with the peak irradiance at 14:30, reaching a value of 60 W.

**Figure 2:**
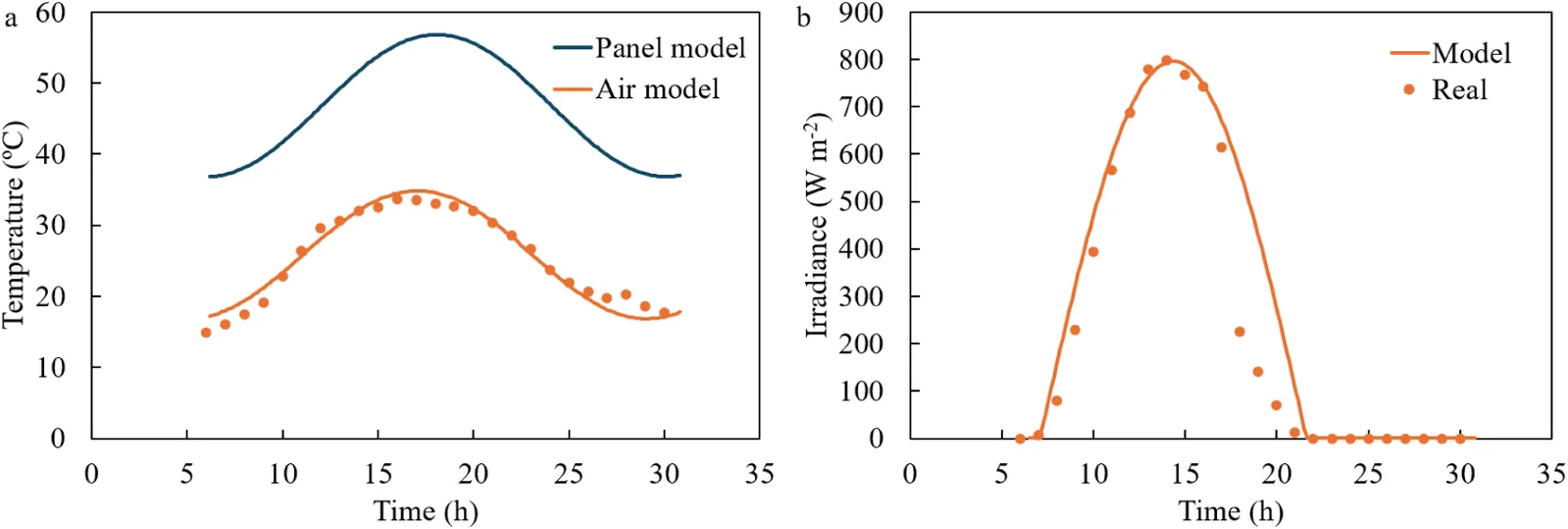
a) Continuous line: temperature of air predicted with eq 7 and on the surface of the panel, predicted with eq S3; and dots: real temperature of air. b) Continuous line: Solar irradiance predicted with eq 6; and dots: real irradiance for the day 27th of May 2025 for Case Study 1.

**Figure 3:**
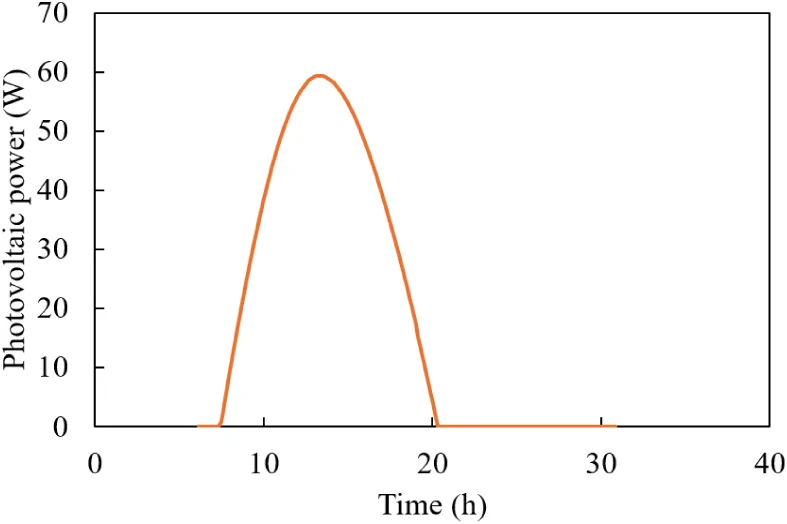
Photovoltaic power generated with a solar panel of 100 W, simulated with eqs 8–10 and eqs S4–S6. Case Study 1, water irrigation pump.

Once the photovoltaic power was determined, the software allocated this energy according to the system’s operating needs. In this case, because the energy demand occurred at night, all photovoltaic power was directed to hydrogen production and storage for later use. This decision logic is shown in Figure S3a, which also depicts the operating scenario. A single panel was active throughout the daytime period, from sunrise until shortly before sunset, continuously producing hydrogen under scenario 1. When irrigation started at 02:00 (corresponding to hour 26 in the plot), the system switched to scenario 3, where the pump was powered by the fuel cell because the stored hydrogen was still sufficient to meet the demand. However, by 04:30, the hydrogen pressure had dropped to a critical level, triggering a transition to scenario 4, in agreement with the profile shown in Figure 5a. The voltage and current of both the electrolyzer and the fuel cell are key parameters, and the simulation model computed these operating conditions along with the corresponding power (eq 11 and eqs S11–S14). Figure 4 shows the predicted profiles. As observed, the electrolyzer operated from sunrise until approximately 20:20, provided that the photovoltaic system supplied enough power. The electrolyzer voltage increased from 4.1 V to a maximum of 5.3 V at 14:30, coinciding with peak solar power; this predicted curve closely matched the experimental data, especially during the last part of the day (Figure 4a). In contrast, the current exhibited greater variability, from 0.42 to 4.7 A, corresponding to the minimum and maximum voltage values dictated by the photovoltaic-power profile (Figure 3). Although the overall trend agreed with the experimental measurements, the discrepancy between predicted and real current values was higher in the maximum of the experimental data (Figure 4b). The fuel cell was activated at night beginning at 02:00, when the system entered scenario 3 (Figure S3). The hydrogen produced during electrolysis (Figure 5) sustained fuel-cell operation for approximately 2 h and 30 min, during which the voltage and current remained constant at 5.2 V and 1.3 A, respectively (eqs S15–S21). Once the stored hydrogen was exhausted, the system switched to the thermal engine (scenario 4). For the fuel cell, the predicted voltage showed very good agreement with experimental data (Figure 4c), whereas the model slightly overestimated the current from 1.3 A predicted versus 1.0 A measured (Figure 4d).

**Figure 4:**
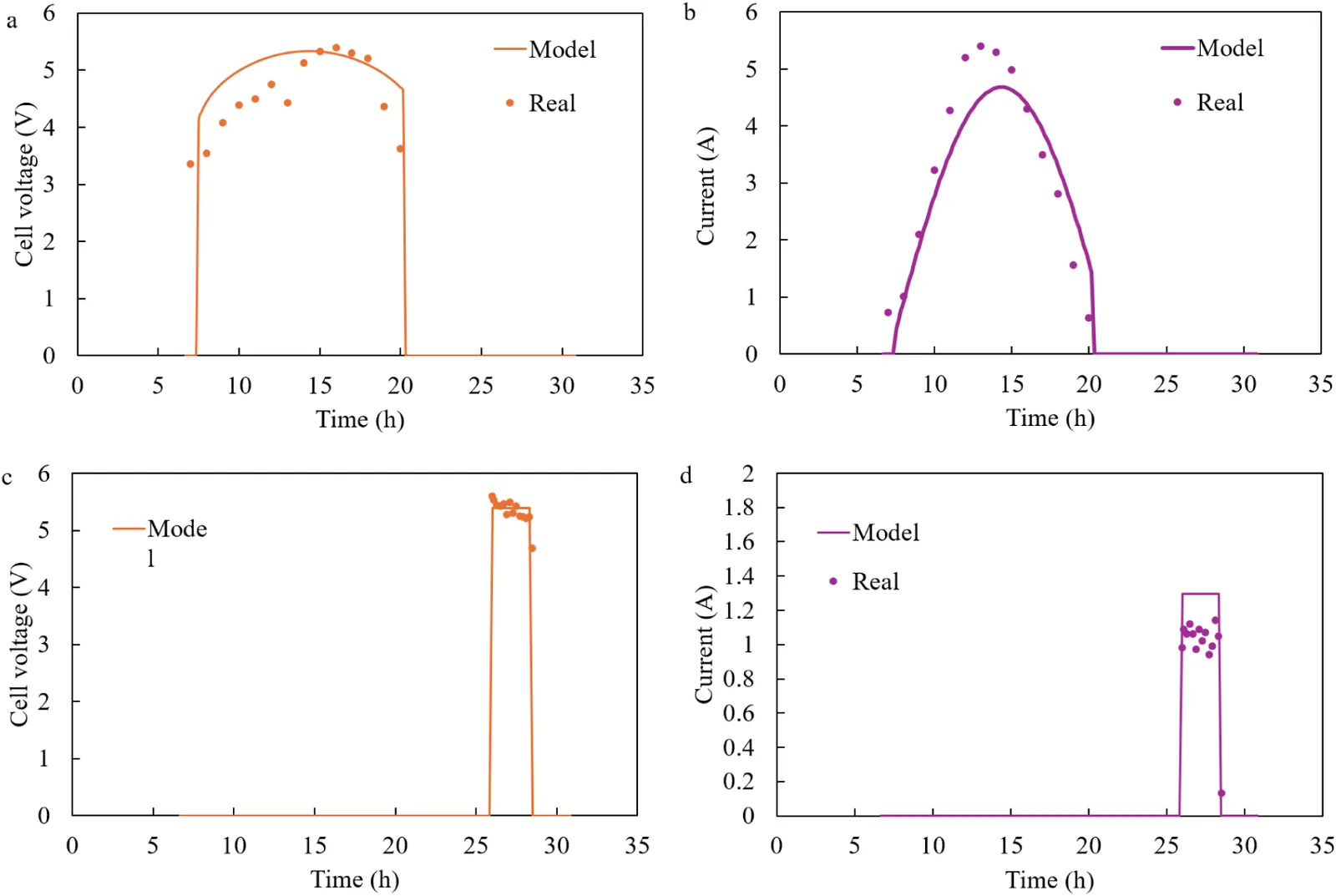
a) Voltage and b) current for the electrolyzer and c) voltage and d) current for the fuel cell, predicted by the model: continuous line, and real data: dots. Case Study 1, water pump system.

**Figure 5:**
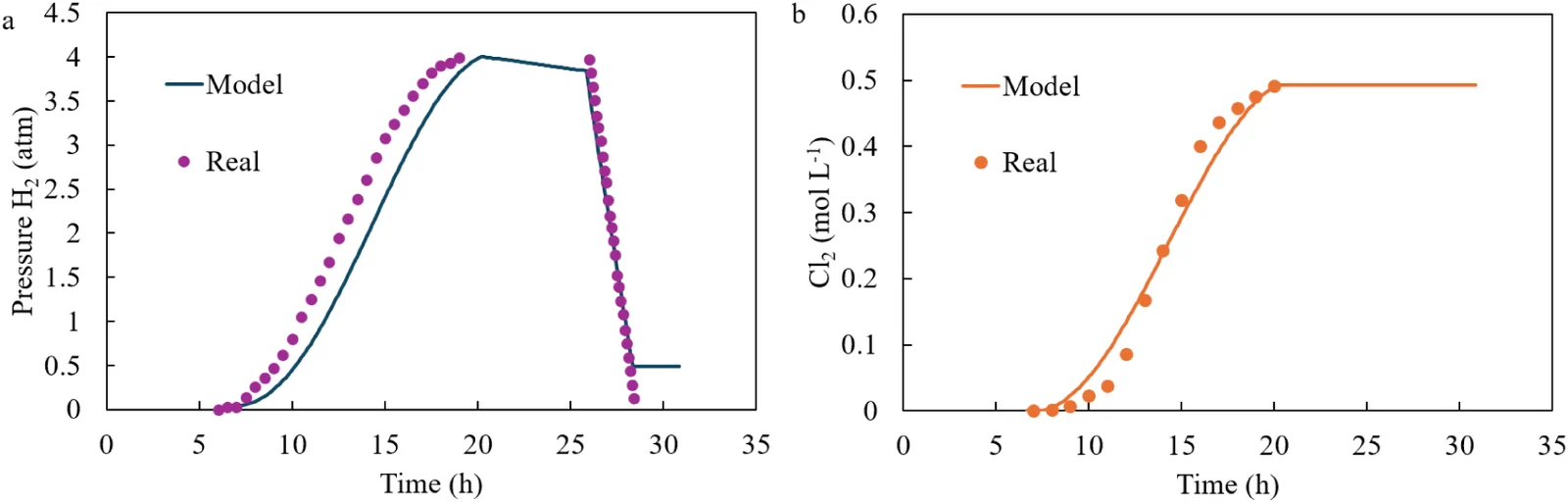
a) H_2_ pressure (atm) and b) chlorine concentration (mol L^–1^); experimental value of Cl_2_ included the gas chlorine but also the devolved in the electrolyte as HClO. Continuous line: model prediction, and dots: real data. Case Study 1, water irrigation pump.

The hydrogen generated and consumed by the electrolyzer and the fuel cell is shown in Figure 5a. In the experiment, the hydrogen was stored in 1-L cylinders pressurized up to 5 bar, with a total of four cylinders filled during the process. For simplicity, the model assumes a single 5-L storage volume. As observed, the total pressure reached 4 bar at approximately 20:30, which corresponds to the same amount of hydrogen stored experimentally in four 1-L cylinders at 5 bar. The model prediction closely matched the experimental data, with only a slight delay during the hydrogen-generation phase. After reaching 4 bar, the pressure decreased gradually until hour 25.5 as a consequence of temperature drop. Once the fuel cell was activated at 02:00, hydrogen pressure began to fall until reaching 0.5 bar, the minimum pressure at which the fuel cell can operate. The model reproduced the hydrogen-consumption profile with high accuracy.

Chlorine produced at the electrolyzer anode increased proportionally, reaching a final concentration of 0.5 mol L^–1^ (Figure 5b). It is important to note that chlorine chemistry is complex and pH-dependent, with several species, such as Cl_2_(*g*), HCl, and HClO, potentially forming in acidic media. For modeling purposes, only molecular chlorine (Cl_2_) was considered to simplify the system. As shown in Figure 6b, the model achieved high accuracy in predicting the anode chlorine production. The values for both hydrogen and chlorine were obtained from mass balances using eqs S27 and S28, respectively.

**Figure 6:**
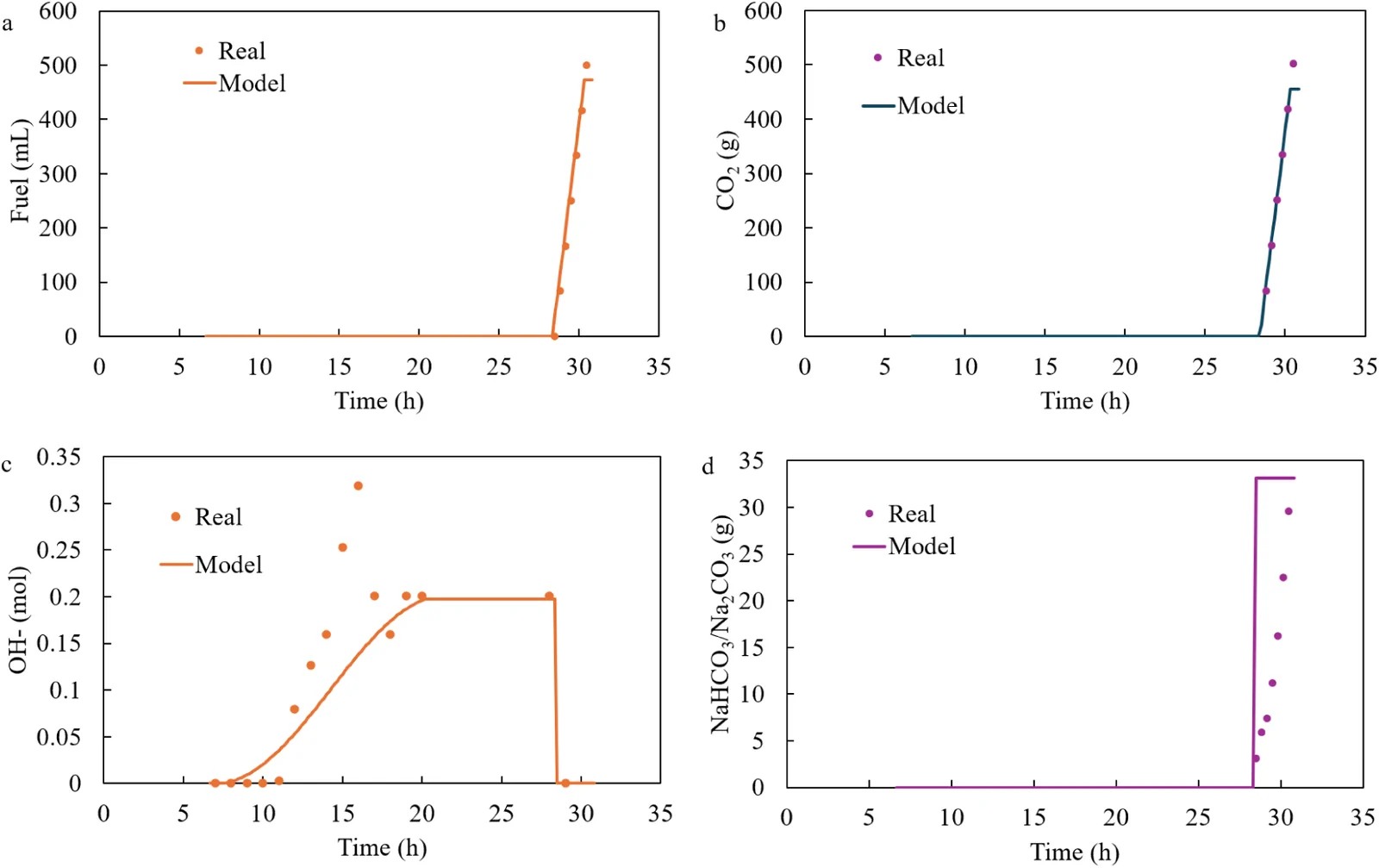
a) Fuel consumption, b) CO_2_ generation, c) hydroxyl generation and consumption and d) carbonates generation by CO_2_ capture with OH^–^ in the cathode solution of the electrolyzer. Continuous line: estimated from model and dots: real data: dots. Case Study 1, water irrigation pump.

The final stage of the experiment occurred when the stored hydrogen was depleted and the thermal engine activated at 04:30. Irrigation continued using the combustion engine, which consumed fuel and produced CO_2_. However, it is important to highlight that the OH^–^ ions generated alongside hydrogen at the electrolyzer cathode were used to partially mitigate CO_2_ emissions. The fuel consumption, CO_2_ generation, OH^–^ production, and subsequent formation of carbonates/hydrogen carbonates are summarized in Figure 6. Figure 6a shows the fuel consumption of the thermal engine, with a maximum reached at hour 30.5 and an excellent fit between model predictions and experimental measurements. The associated CO_2_ generation is presented in Figure 6b, also showing very strong agreement between predicted and real values. As previously explained, hydroxyl ions were included in the model because they were responsible for capturing CO_2_. As shown in Figure 6c, the moles of OH^–^ increased to a maximum of approximately 0.2 mol. This value is lower than the theoretical amount expected if electrolyzer efficiency approached unity (as was the case for H_2_). This discrepancy can be attributed to acid formation in the anode compartment, specifically HCl or HClO, arising from the disproportionation of dissolved Cl_2_. The resulting H^+^ ions could migrate through the membrane and neutralize part of the OH^–^ generated at the cathode. In the experimental data, a sharp increase in OH^–^ at around hour 15 is followed by a decline and stabilization near 0.2 mol; this behavior is consistent with the neutralization mechanism described above. Finally, due to the reaction between CO_2_ and OH^–^, sodium carbonate (Na_2_CO_3_)/hydrogen carbonate (NaHCO_3_) was formed, as shown in Figure 6d. A slight deviation between model predictions and experimental data is observed, likely because the model assumed instantaneous carbonate/bicarbonate formation, whereas the actual reaction may proceed at a slower rate or even problems related to mass transfer limitations.

The final component of the analysis, following the mass-balance evaluation, was the assessment of the energy generated or consumed by each device; all results are presented in Figure 8. The total solar-energy production for the full day was nearly 500 Wh; however, the electrolyzer received only about 200 Wh. As shown in Figure 7a, the model predicted the actual energy supplied to the electrolyzer with high accuracy. The energy associated with the remaining devices (the fuel cell, the thermal engine, and the water pump) is summarized in Figure 7b. The model slightly overestimated the energy generated by the fuel cell, which can be attributed to the small overprediction of voltage and current observed previously (Figure 4c–d). For the thermal engine and the water pump, the agreement between model predictions and experimental data was better: both the fuel cell and the thermal engine delivered approximately 14 Wh, while the water pump consumed 28 Wh.

**Figure 7:**
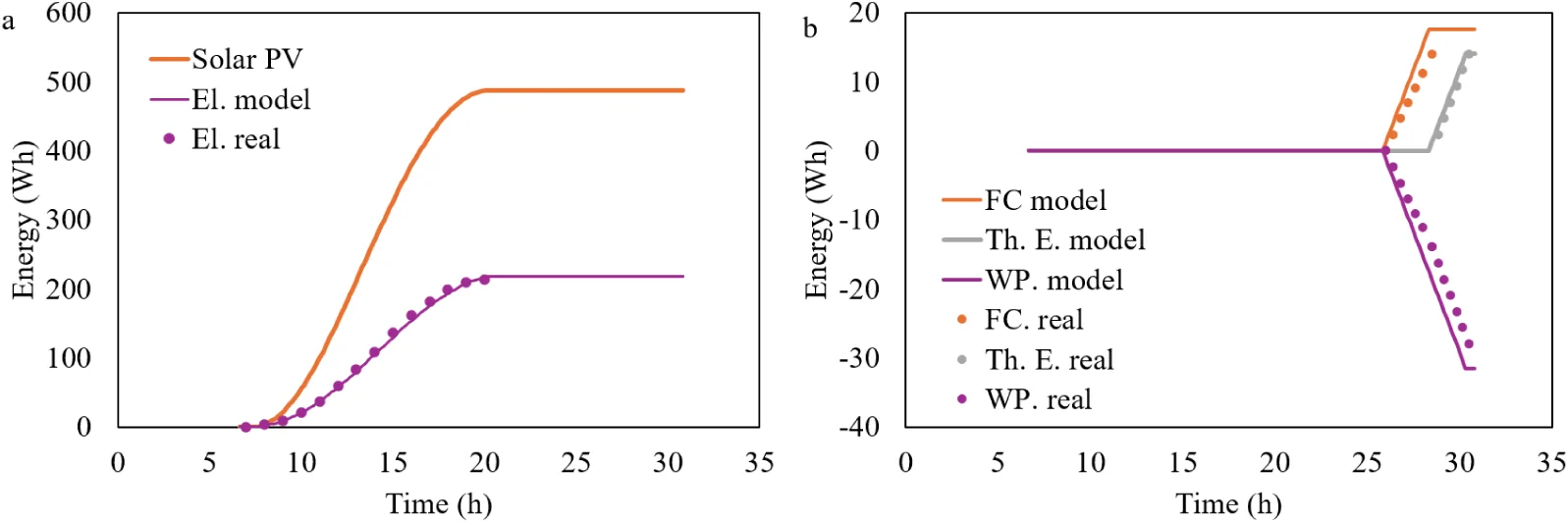
Energy consumption and generation for all the systems involved in the simulation process: a) Photovoltaic panel and electolyzer (El.), b) fuel cell (FC.), thermal engine (Th. E.) and water pump (WP). Continuous line: model simulations and dots: real data from experimental setup. Case Study 1, water irrigation system.

### 3.2 Case Study 2

The second case study presents some peculiarities that make the simulation more difficult and, consequently, more interesting.^18^ In this case, all the systems could be activated during the day because a delivery vehicle was simulated. It was charged (producing H_2_) at night using grid electricity due to the lower price. The vehicle was used four times during the day at the following time intervals: 7:00–7:30, 15:30–16:00, 19:30–20:00, and 21:30–22:00. These intervals were selected to evaluate the performance of the prototype under different real operating conditions and working modes.

The temperature and solar radiation profiles showed similar patterns to those in the previous case study, as can be seen in Figure S4. The data predicted by the model reproduced the real measurements with high accuracy.

In this case, the electrolyzer operated with grid electricity from 00:00 to 06:00 (scenario 5, Figure S3), during which the hydrogen tank reached its maximum pressure (Figure 9a). At 07:00, the vehicle started moving, and the fuel cell was supplied with hydrogen stored during the night (scenario 3, Figure S3). The operating conditions of these devices are shown in Figure 8. The electrolyzer voltage (Figure 8a) and current (Figure 8b) were accurately reproduced, whereas the error in the fuel cell voltage (Figure 8c) was higher. Once the vehicle stopped, solar irradiance was sufficient to power the electrolyzer and produce hydrogen again (Figure 9, scenario 1, Figure S3), and the cell voltage and current were once more well fitted by the model (Figure 8a and 8b, respectively). At 15:30, the vehicle moved again; however, in this case, all the consumption was covered by solar panels, with no hydrogen usage (scenario 2, Figure S3). The next movement occurred at 19:30. As sunset was approaching, irradiance was low (Figure S4), and the electric engine was powered by the fuel cell again (scenario 3, Figure S3), with a much better prediction accuracy from the model (Figure 8c and 8d). Hydrogen was consumed again, and the electrolyzer resumed operation until sunset (scenario 1, Figure S3); in this period, the predicted current was underestimated (Figure 8a). Finally, at 0:00 (24 h), the car was fast-charged using grid electricity (scenario 5, Figure S3). As shown in Figure 8a and 8b, the model predicted the data with lower accuracy at this stage, overestimating the current and underestimating the cell voltage. This effect may be associated with a poorer model adjustment at high currents compared to lower ones. The error at high currents was associated with the data used to calibrate of the model (1.8, 3.6, and 5.4 A).^11^

**Figure 8:**
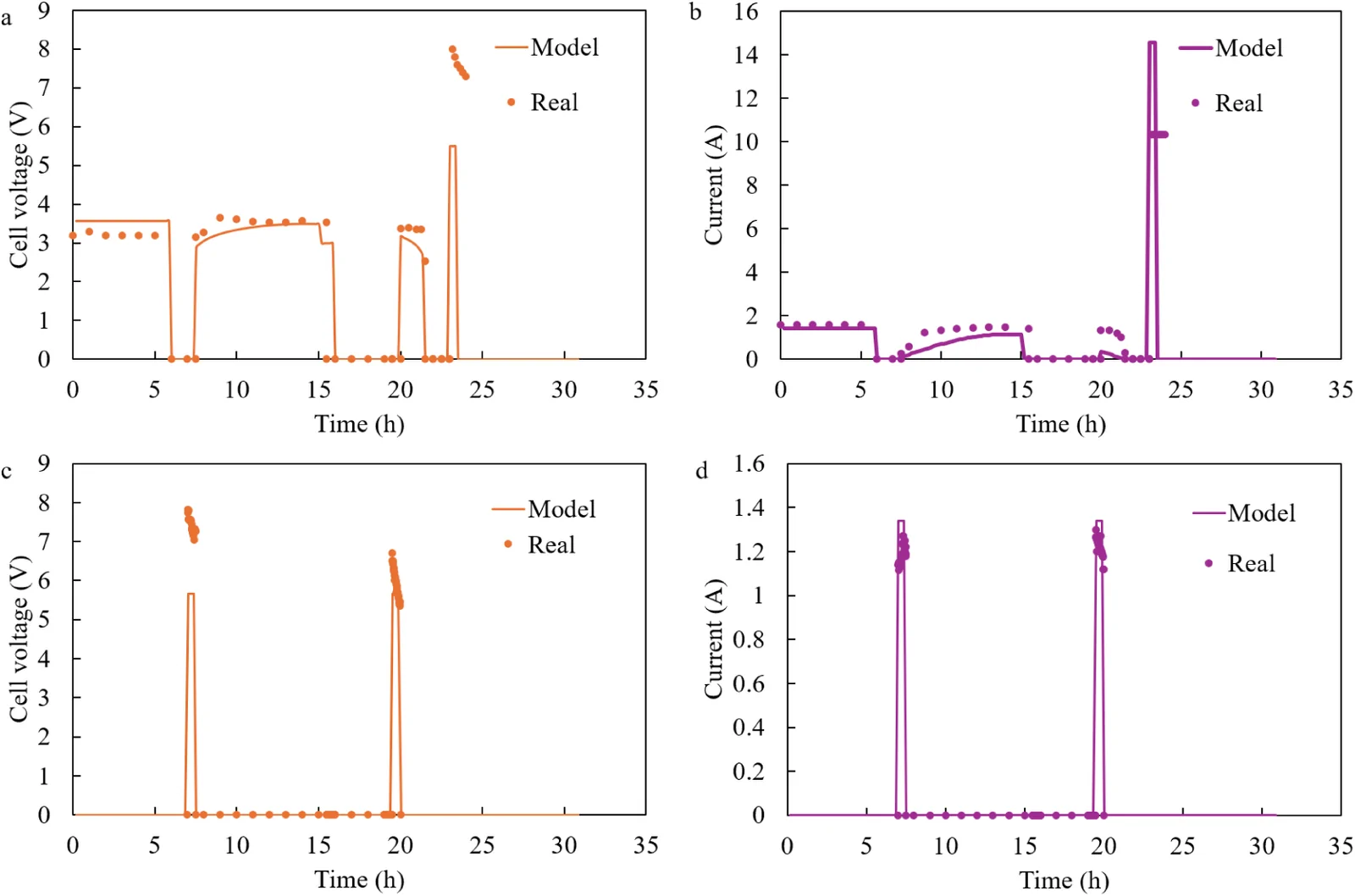
a) Voltage and b) current for the electrolyzer, and c) voltage and d) current for fuel cell, predicted by the model: continuous line; and real data: dots. Case Study 2, hybrid car.

**Figure 9:**
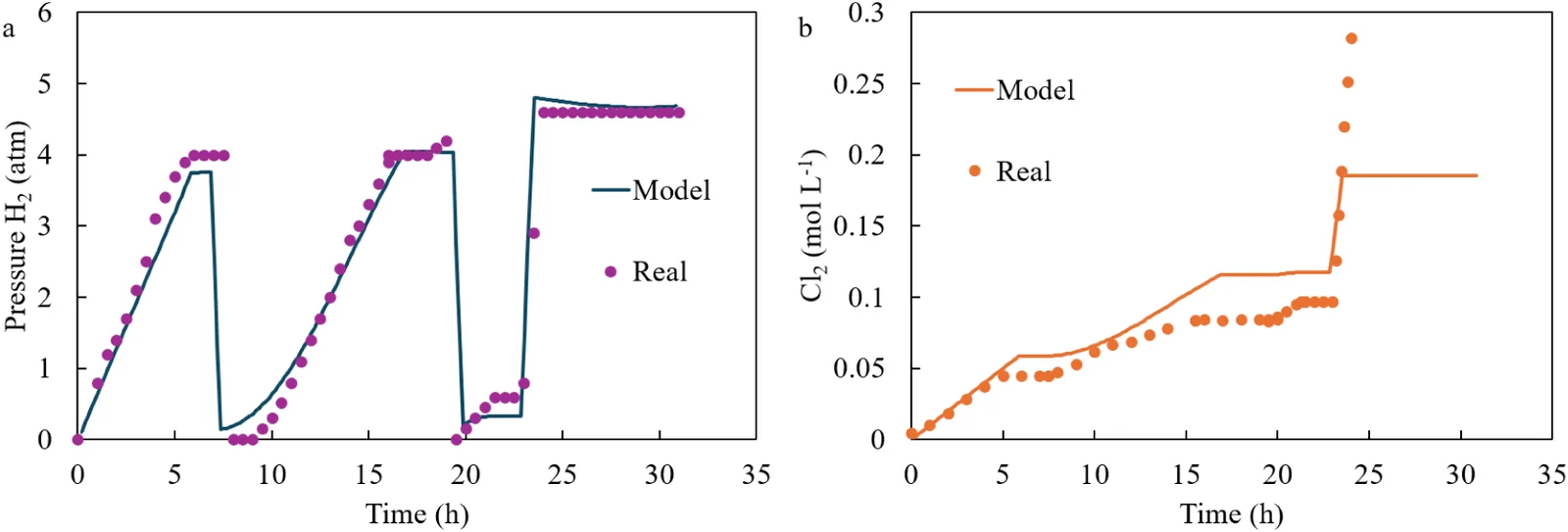
a) H_2_ pressure (atm) and b) chlorine concentration (mol L^–1^); experimental value of Cl_2_ included the gas chlorine but also the devolved in the electrolyte as HClO. Continuous line: model prediction, and dots: real data. Case study 2, hybrid vehicle.

The H_2_ and Cl_2_ generated and consumed during the process described above are shown in Figure 9. As in Case study 1 (Figure 5a), the model exhibited very high accuracy in predicting H_2_ behavior (Figure 9a). In this case, there was a slight difference in the maximum pressure reached: 4 bar in the experiment and 3.8 bar in the model, as well as in the hydrogen consumption by the fuel cell. On both occasions when the fuel cell was activated, the stored H_2_ was completely consumed, whereas the model predicted a remaining pressure of 0.1 bar.

Regarding chlorine concentration (Figure 9b), the prediction was highly accurate throughout the entire process except during the final fast-charging step. In this stage, the amount of Cl_2_ produced experimentally was higher than the value predicted by the model, which may be related to a change in the efficiency of the chlorine reduction reaction at high current densities.^19^

Regarding the thermal engine stage, fuel consumption and CO_2_ generation were perfectly predicted by the model, with no observable differences (Figure 10a and 10b). In contrast, hydroxyl generation (Figure 10c) was not accurately predicted, which may be related to the neutralization of OH^–^ by the H^+^ produced at the electrolyzer anode, as explained in Case study 1. The carbonates/bicarbonates formed during the carbon capture process also showed a high prediction error (Figure 10d), this could be associated with a low efficiency in the carbon capture process. In the real test with the prototype only 1% of the total CO_2_ generated was captured. This effect was associated with the bad mass transfer taking place in the electrolyzer more than the kinetic of the process.^11^

**Figure 10:**
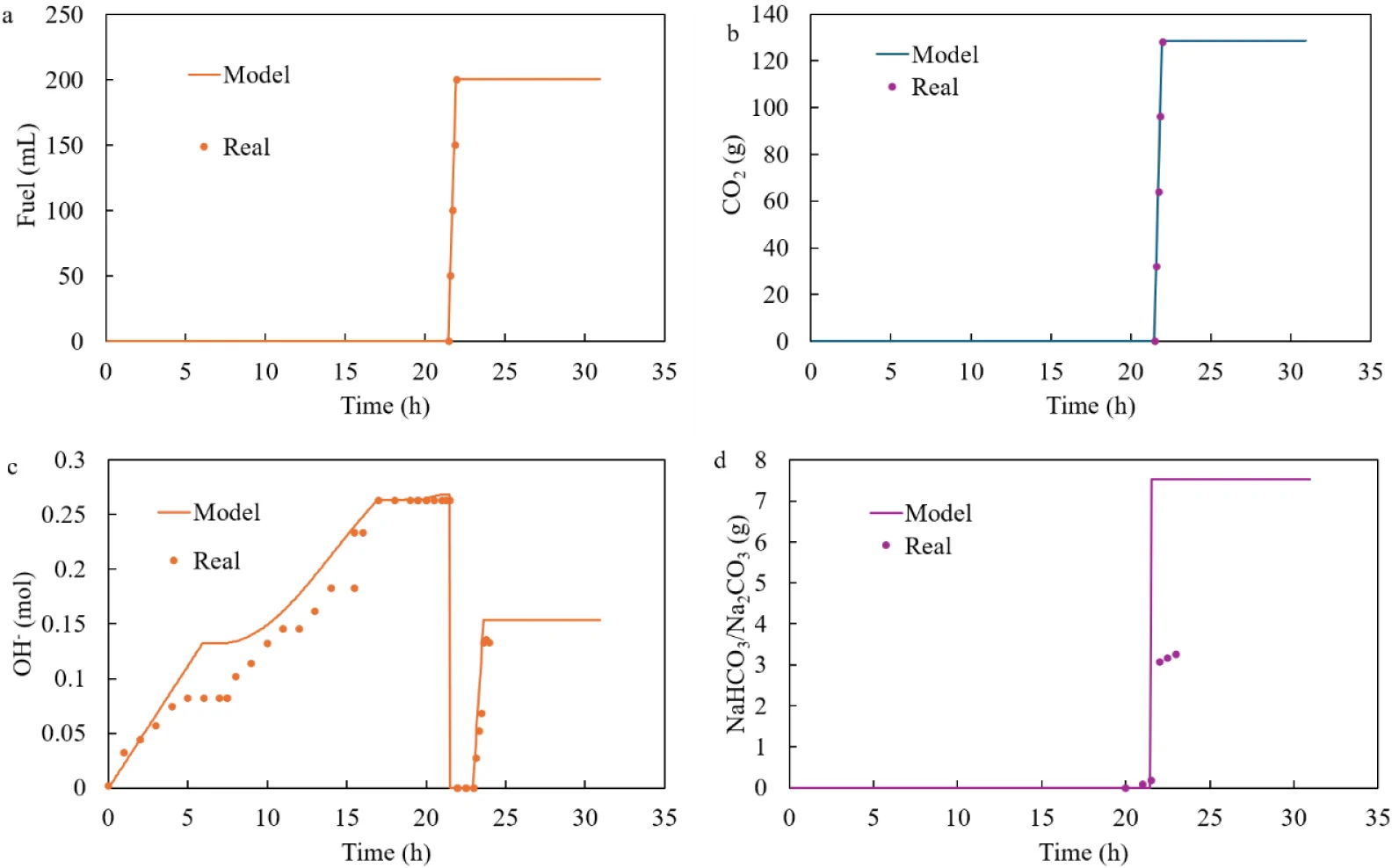
a) Fuel consumption, b) CO_2_ generation, c) hydroxyl generation and consumption and d) carbonates generation by CO_2_ capture with OH^–^ in the cathode solution of the electrolyzer. Continuous line: estimated from model and dots: real data: dots. Case Study 2, hybrid vehicle.

Finally, the energy balances were analyzed for all the equipment (Figure 11). The highest error in this case corresponded to the electrolyzer, as shown in Figure 11a. The model underestimated the energy consumed by this system compared to the experimental values. It had previously been determined that there were differences between the real and predicted cell voltage (Figure 8a and 8b) and current for this device, which could explain the discrepancies, particularly during the fast-charging stage. For the rest of the systems, the prediction accuracy was much higher, with the modeled data showing no observable differences from the experimental results (Figure 11b).

**Figure 11:**
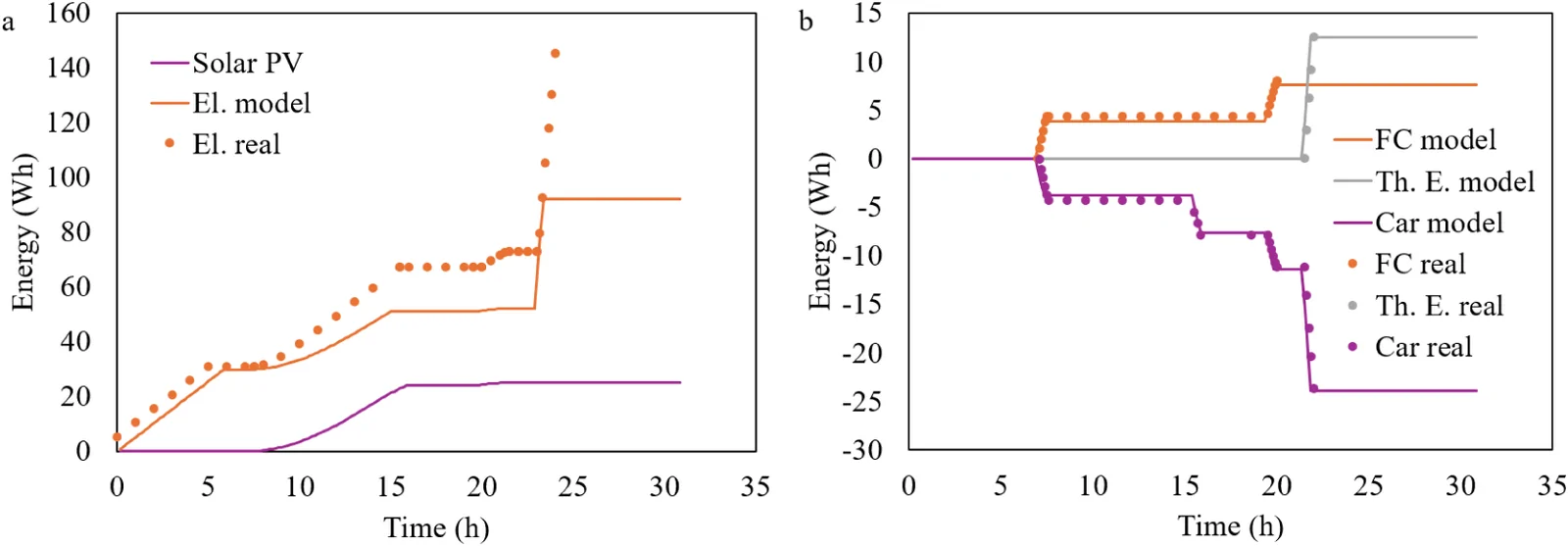
Energy consumption and generation for all the systems involved in the simulation process: a) Photovoltaic panel and electolyzer (El.), b) fuel cell (FC.), thermal engine (Th. E.) and water pump (WP). Continuous line: model simulations and dots: real data from experimental setup. Case Study 2, hybrid vehicle.

### 3.3 Error of the Model and Sensitivity Analysis

The goodness of fit of the models is shown in Figure 12. Figure 12a presents the modeled values versus the experimental values, with both quantities normalized by dividing them by the mean experimental value (both case studies were included). The energy model aligns closely with the diagonal, indicating an excellent fit, whereas for matter the lower values exhibit a much better agreement than the higher ones. This was also confirmed in Figure 12b, where the residuals were plotted against the experimental values, with both quantities normalized in the same manner. As can be observed, for the lower values the point cloud lies very close to zero, as expected for a good fit. However, for higher values in the case of matter, the residuals increase slightly and deviate downward, indicating a poorer fit for these data, which is consistent with the larger discrepancies observed in the prediction of Cl_2_ or NaHCO_3_/Na_2_CO_3_ in Case study 2. Finally, the R-square was calculated for the matter and energy adjustments using eqs S38–S40. The resulting values were 0.926 and 0.981, respectively, confirming the good quality of the model fit for predicting the data in case studies 1 and 2.

**Figure 12:**
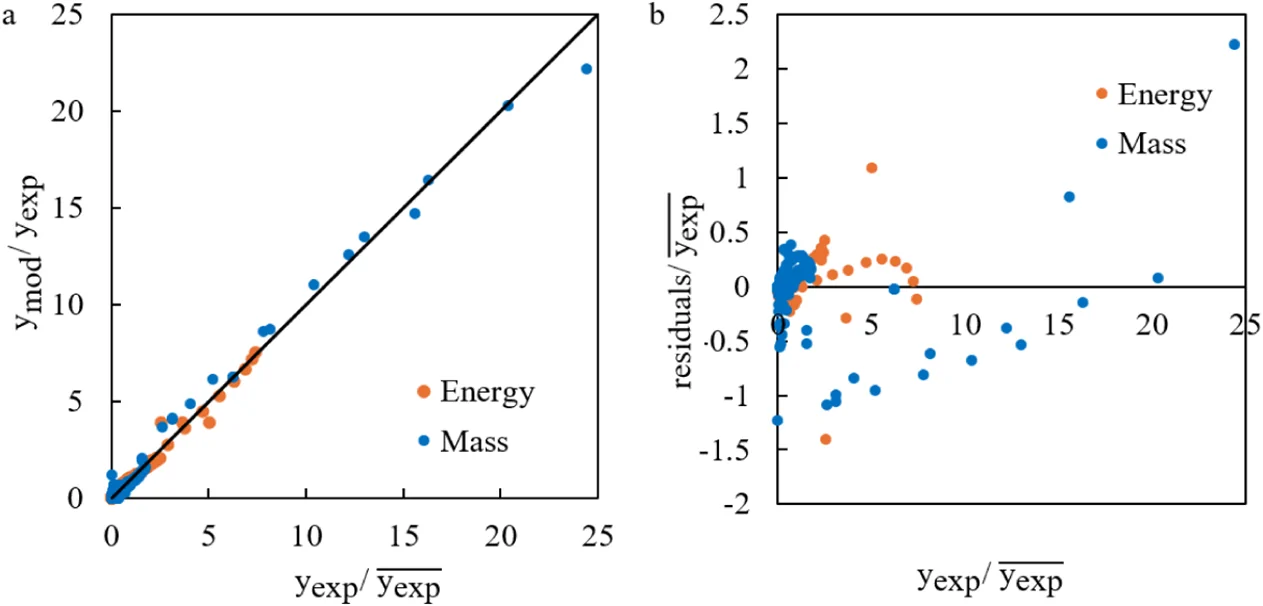
a) Modeled data versus experimental data, both normalized by dividing them by the mean experimental value. b) Residuals versus the experimental value, also normalized by the mean experimental value. Data from both case studies are included. Energy is shown in orange and matter in blue.

Additionally, the mean absolute error (MAE, eq S41) and the root-mean-square error (RMSE, eq S42) were calculated for each variable in both case studies. The results are presented in Table 2. It can be observed that the MAE and RMSE values were very low for H_2_, Cl_2_, and OH^–^ in both case studies. However, in Case Study 1, higher errors were obtained for fuel, CO_2_, and bicarbonate. This may be attributed to the limited number of experimental data points, which hinder accurate model predictions. In Case Study 2, the discrepancies were lower for all variables, with the highest MAE being 0.19 mol for fuel and the highest RMSE being 0.27 mol. Overall, the model exhibited better accuracy in Case Study 2 than in Case Study 1.

**Table 2:** Mean Absolute Error, MAE; and Root Mean Square Error, RMSE Calculated for the Different Variables of the Models for the Two Cases of Study: Case Study 1, Water Irrigation and Case Study 2, Hybrid Vehicle

	**Case Study 1**		**Case Study 2**	
	MAE	RMSE	MAE	RMSE
**Mass (mol)**				
H_2_	0.07	0.08	0.02	0.05
Cl_2_	0.02	0.02	0.02	0.02
OH^–^	0.04	0.06	0.02	0.02
Fuel	0.41	0.42	0.19	0.27
CO_2_	0.29	0.44	0.17	0.25
Carbonate	0.15	0.17	0.05	0.07
**Energy (Wh)**				
Electrolyzer	2.96	3.92	6.73	11.17
Fuel Cell	2.82	3.03	0.42	0.51
Thermal Engine	1.00	1.08	0.73	1.12
Consumption	3.83	3.99	1.44	2.19

In terms of energy, the obtained error values were low in all cases, remaining below 3 for almost all variables. The highest error was associated with the electrolyzer in Case Study 2, reaching an MAE of 6.73 and an RMSE of 11.17. These high values were mainly attributed to the poor accuracy of predictions at high current levels.

A sensitivity analysis was also performed to verify that the selected time step (Δ*t* = 600s) provided sufficient accuracy. The corresponding root mean squared relative error (RMSRE) was calculated using eq S43. The results are presented in Figure 13. The analysis showed that, for most of the evaluated variables, the relative error associated with time steps smaller than 600 s was negligible and close to zero, except for H_2_ in Case Study 2, where it reached approximately 10%. However, when the time step was increased to 1200 or 2400 s, the relative error increased significantly in Case Study 1, exceeding 5% for most variables and reaching up to 20% at Δ*t* = 1200 s (Figure 13a). In Case Study 2 (Figure 13b), which considered the hybrid vehicle, the effect of increasing the time step was even more pronounced. At Δ*t* = 1200 s, the RMSRE increased from approximately 3% at Δ*t* = 600 s to more than 20% for several variables, exceeded 35% for fuel consumption and CO_2_ emissions, and reached values as high as 200% for H_2_ consumption. Further increasing the time step to 2400 s resulted in an additional deterioration of the model accuracy.

**Figure 13:**
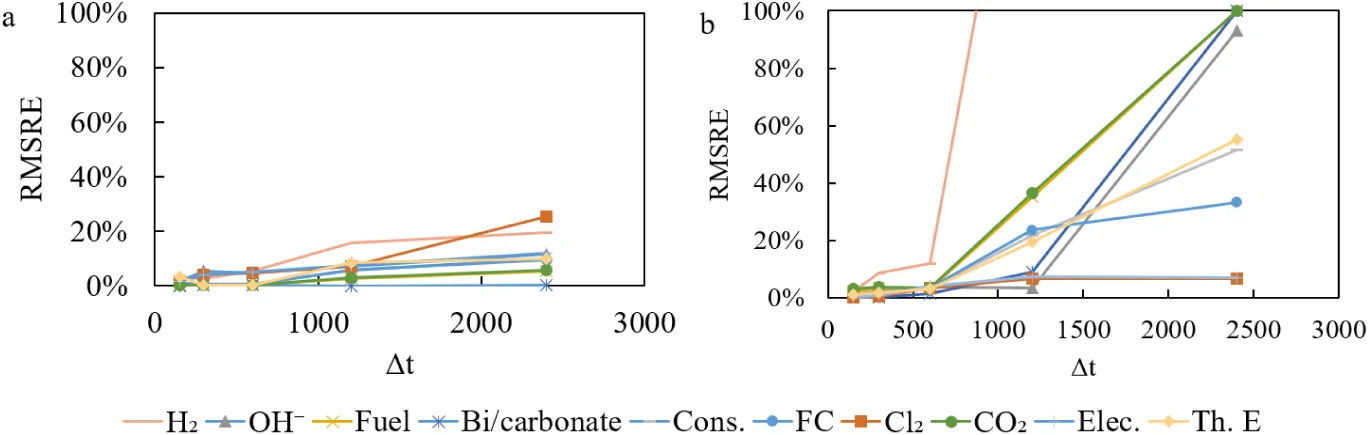
Root mean relative square error, RMSRE for the sensitivity analysis carried out with Dt of 150, 300, 600, 1200, and 2400 s for a) Case study 1, water irrigation pump, and b) Case study 2, hybrid vehicle.

## 4 Conclusions

To the best of our knowledge, this is the first model where a chloralkaline process is integrated with solar photovoltaic panels for H_2_ production and their application in two different scenarios with the addition of carbon capture in the same procedure. The following conclusions can be drawn:

1. It is feasible to develop a simple simulator capable of accurately reproducing the behavior of a solar-hybrid engine and to implement it in a spreadsheet environment by integrating four models: photovoltaic energy generation, electrochemical energy storage, engine energy supply, and engine operation. These models are solved using a first-order Euler discretization across several operating scenarios that govern the functioning of the solar-hybrid engine.
2. The parameters of the different models can be readily determined by fitting time-series models to environmental data collected at the application site, adjusting solar-panel behavior according to the technical specifications provided by manufacturers, and performing straightforward experiments in which the behavior of each subsystem is isolated.
3. The model successfully reproduces the behavior of hybrid engines implemented in agricultural irrigation and automotive prototypes, achieving correlation coefficients of 0.981 for energy-related data and 0.926 for the concentration and quantity of the chemical species involved. RMSE and MAE confirmed the goodness of model estimations pointing out the existence of deviations for some parameters.
4. The good agreement between model predictions and experimental results supports the potential use for the future use of this tool in energy-management systems for this type of hybrid engine.

However, there were also some drawbacks, the model could reproduce the experimental data with enough accuracy, however, some defects were detected. The most important one was related to the electrolyzer when high currents were used, a wider range of calibration data would be helpful. The chlorine production also failed in some cases. And finally, the carbon capture, due to the low efficiency in the real scenarios, maybe the model could not reproduce it with high accuracy.

## Supplementary Material



## Notes

Detailed experimental procedures, equations used in the mathematical model description, and additional figures, including screenshots of the model, the working mode of the model, and additional results from the simulation. There is also a table with the technical data used for the simulation of solar photovoltaic panels (PDF)
