# Supplementary material for: Smart Simulation Solutions for Hybrid Solar Engine Performance
Source: ACS Omega. 2026 Oct 20;11(43):67339–51. doi: 10.1021/acsomega.6c08338 (PMC13736870; doi:10.1021/acsomega.6c08338)
Supplement: Supplementary file 1 [file ao6c08338_si_001.pdf]

Smart Simulation Solutions for Hybrid Solar Engine Performance

Miguel A. Rodriguez-Cano, Iñaki Requena-Leal, Justo Lobato, Manuel A. Rodrigo

Department of Chemical Engineering, Faculty of Chemical Sciences & Technologies. Universidad de Castilla-La Mancha, Ciudad Real 13071, Spain

Supplementary information

S1. Formulation of models

Photovoltaic energy-generation model.

The moments of sunrise and sunset are modeled according to Eqs. S1 and S2, where $t_{sunrise}$ and t_{sunset} represents the hour of sunrise and sunset respectively in hours for the day of the year n_{day}. This model uses the sunrise hour for summer and winter solstice, $t_{sunrise,min}$ and $t_{sunrise,max}$ respectively, and the sunset hour for the same days, $t_{sunset,min}$ for winter solstice and $t_{sunset,max}$ for summer solstice, all of them in hours (0-24). There are also two experimental parameters φ_1 and φ_2 with values of -80.75 and -264.65.

$$t_{sunrise} = t_{sunrise,min} + \left(t_{sunrise,max} - t_{sunrise,min}\right) \cdot \frac{1}{2} \cdot \sin\left(\frac{2 \cdot \pi \cdot (n_{day} + \varphi_1)}{365}\right) \quad [Eq. S. 1]$$

$$t_{sunset} = t_{sunset,min} + \left(t_{sunset,max} - t_{sunset,min}\right) \cdot \frac{1}{2} \cdot \sin\left(\frac{2 \cdot \pi \cdot (n_{day} + \varphi_2)}{365}\right) \quad [Eq. S.2]$$

The temperature at the surface of the photovoltaic solar panel is calculated using Eq. S3, where $T_{p,min}$ and $T_{p,max}$ corresponded to the maximum and minimum temperatures (K) on the surface of the panel for the selected day, n_{day}

$$T_p\ (K) = T_{p,min} + \left(T_{p,max} - T_{p,min}\right) \cdot \frac{1}{2} \cdot \left(1 + \sin\left(\frac{2 \cdot \pi \cdot (n_{day} + \varphi_3)}{365}\right)\right) \cdot \left(\sin\left(\frac{0.7 \cdot \pi}{t_{sunset} - t_{sunrise}}\right) \cdot \left(\frac{t}{3600} - t_{sunset} - 5\right)\right) \quad [Eq. S.3]$$

The parameters of the solar panels are determined using Eqs. S4 to S6, where I_{SC} denoted the short circuit current (Table S.1); K_I, the temperature coefficient; λ, the solar radiation (Eq. 1) and λ_{ref} the reference solar radiation (Table S.1); E_{GO}, the band gap for silicon (1.22 eV); and, V_{OC} represented the open circuit potential (Table S.1). For the integration of multiple panels, Eqs. S7 to S14 are used, where P_{PV} is power delivered by one solar panel, as a function of the current, I_{PV}, and the voltage, V_{PV}. N_{PV}^{P} and N_{PV}^{S} represented the number of panels in parallel and series. I_{PV}^{P} corresponded to the sum of the current for the panels in parallel and V_{PV}^{S} to the sum of voltage for the panels in series, being P_{PV}^{matrix} the total power of the matrix of panels in series and/or parallel. j_{el} represented the current density of the electrolyzer, first approached as a function of the current of the solar panel, I_{PV}; and the area of the electrodes of the electrolyzer, $area_{el}$. Er_{el} represented the error for the current density estimated and the calculated one for the electrolyzer module. I_{el} corresponded to the current in the electrolyzer module and P_{el} to its power.

$$I_{ph} = I_{SC} + K_I \cdot (T_p - 298) \cdot \frac{\lambda}{\lambda_{ref}} \; [Eq. S.4]$$

$$I_s = I_{rs} \cdot \left(\frac{T_p}{298}\right)^3 \cdot e^{\left(\frac{q \cdot E_{G0}}{n \cdot K_{Boltzmann}}\right) \cdot \left(\frac{1}{298} - \frac{1}{T_p}\right)} \; [Eq. S.5]$$

$$I_{rs} = \frac{I_{SC}}{e^{\left(\frac{q \cdot V_{OC}}{N_C \cdot n \cdot K_{Boltzman} \cdot T_p}\right)} - e^{(I_{SC} \cdot R_S)}} \; [Eq. S.6]$$

.

$$P_{PV} = I_{PV} \cdot V_{PV} \; [Eq.\ S.7]$$

$$I_{PV}^P = N_{PV}^P \cdot I_{PV} \; [Eq.\ S.8]$$

$$V_{PV}^S = N_{PV}^S \cdot V_{PV} \; [Eq.\ S.9]$$

$$P_{PV}^{matrix} = I_{PV}^P \cdot V_{PV}^S \; [Eq.\ S.10]$$

$$j_{el} = \frac{I_{PV} \cdot 0.5 \cdot 1000}{area_{el}} \; [Eq.\ S.11]$$

$$Er_{el} = j_{el} - \frac{\left(\frac{P_H}{V_{el}}\right)}{area_{el}} \cdot 1000 \; [Eq.\ S.12]$$

$$I_{el} = j_{el} \cdot area_{el} \; [Eq.\ S.13]$$

$$P_{el} = V_{el} \cdot I_{el} \; [Eq.\ S.14]$$

Engine powering model. For modeling the fuel cell, Eqs. S15 to S20 are used, whereP_{FC} was the power generated by the fuel cell, based on V_{FC}, the voltage and I_{FC} the current, determined after an optimization (minimization of the error, Er_{FC}) of the current density j_{FC} estimated; $area_{FC}$ corresponded to the area of the electrodes of the fuel cell. For modeling the combustion engine, Equation S.21 was used where P_F corresponde to the power that the thermal engine must supply.

$$P_{FC} = P_{cons} - P_{PV}^{matrix} \quad [Eq.\ S.15]$$

$$V_{FC} = a_{FC} + b_{FC} \cdot Log(j_{FC}) + c_{FC} \cdot j_{FC} \quad [Eq.\ S.16]$$

$$Er_{FC} = j_{FC} - \frac{\left(\frac{P_{FC}}{V_{FC}}\right)}{area_{FC}} \cdot 1000 \quad [Eq.\ S.17]$$

$$j_{FC} = \frac{\left(\frac{P_{FC}}{V_{FC}}\right)}{area_{FC}} \cdot 1000 \quad [Eq.\ S.18]$$

$$I_{FC} = j_{FC} \cdot area_{FC} \quad [Eq.\ S.19]$$

$$P_{FC} = V_{FC} \cdot I_{FC} \quad [Eq.\ S.20]$$

$$P_{F} = P_{cons} - P_{PV}^{matrix} \quad [Eq.\ S.21]$$

Electrochemical energy storage & Engine performance models. The energy balances are calculated according to Eqs. S22 to S26.

$$E_{PV}^{t} = E_{PV}^{t-\Delta t} + P_{PV}^{matrix} \cdot \frac{\Delta t}{3600} \quad [Eq.\ S.22]$$

$$E_{el}^{t} = E_{el}^{t-\Delta t} + P_{el} \cdot \frac{\Delta t}{3600} \quad [Eq.\ S.23]$$

$$E_{FC}^{t} = E_{FC}^{t-\Delta t} + P_{FC} \cdot \frac{\Delta t}{3600} \quad [Eq.\ S.24]$$

$$E_{F}^{t} = E_{F}^{t-\Delta t} + P_{F} \cdot \frac{\Delta t}{3600} \quad [Eq.\ S.25]$$

$$E_{cons}^{t} = E_{cons}^{t-\Delta t} + P_{cons} \cdot \frac{\Delta t}{3600} \quad [Eq.\ S.26]$$

The mass balances are applied to the most relevant species, including hydrogen, chlorine, fuels, hydroxide ions, and carbon dioxide. The hydrogen balance is given in Eq. S27, where the amount of hydrogen at time $t - \Delta t$ was denoted as $H_2^{t-\Delta t}$. The rest of parameters were Q_{H_2} for the volumetric flow; p, represented the pressure of the system; R the ideal gas constant, and T the ambiance temperature (Eq. 4). The output H_2 moles were $mol_{H_2}^{out}$. The current of the electrolyzer, I_{el}, and the fuel cell, I_{FC}, were estimated with Eq. 19 and 25, respectively. Finally, F represented the faradaic constant and $e_{H_2}^{el}$ and $e_{H_2}^{FC}$ where the hydrogen efficiency in the electrolyzer and the fuel cell respectively. The balance of chlorine is shown in equation S28, where $[Cl_2]^t$ represented the concentration in mol L^{-1} at time $t - \Delta t$, Vol_{ano} was the volume of the anolyte, $Q_{anol,el}$ the volumetric flow of the anolyte in the electrolyzer; $[Cl_2]_{in}^{t}$, the concentration of chlorine at the entrance of the electrolyzer at time t; and $e_{Cl_2}^{el}$ the efficiency of chlorine in the electrolyzer. The concentration of hydroxyl anions at time t, $[OH^-]^t$ was calculated with Eq. S.29, depending on the concentration at time, $t - \Delta t$, $[OH^-]^{t-\Delta t}$; the volume of catholyte, Vol_{cat}; the volumetric flow rate of catholyte, $Q_{anol,el}$; the inflow concertation of hydroxyl at time t, $[OH^-]_{in}^{t}$. I_{el} was the current in the electrolyzer and $e_{H_2}^{el}$ the efficiency of the electrolyzer cathode. $OH^{-\,t}$ represented the mol of hydroxyl at time t calculated with Eq. S.30 as a function of the quantity at time $t - \Delta t$, $OH^{-\,t-\Delta t}$ and the quantity generated in the cathode of the electrolyzer.

$$H_2^{t} = H_2^{t-\Delta t} + \Delta t \cdot \left(\frac{Q_{H_2} \cdot p}{R \cdot T} - mol_{H_2}^{out} + \frac{I_{el}}{2 \cdot F \cdot e_{H_2}^{el}} - \frac{I_{FC}}{2 \cdot F} \cdot e_{H_2}^{FC} \right) \quad [Eq.\ S.27]$$

$$[Cl_2]^{t} = [Cl_2]^{t-\Delta t} + \frac{\Delta t}{Vol_{ano}} \cdot \left(Q_{anol}^{el} \cdot ([Cl_2]_{in}^{t} - [Cl_2]^{t}) + \frac{I_{el}}{2 \cdot F \cdot e_{Cl_2}^{el}} \right) \quad [Eq.\ S.28]$$

$$[OH^-]^t = [OH^-]^{t-\Delta t} + \frac{\Delta t}{Vol_{cat}} \cdot \left(Q_{cat}^{el} \cdot ([OH^-]_{in}^t - [OH^-]^t) + \frac{I_{el}}{F \cdot e_{H_2}^{el}} \right) \quad [Eq.\ S.29]$$

$$OH^{-\,t} = OH^{-\,t-\Delta t} + [OH^-]^t \cdot Vol_{cat} \ [Eq.\ S.30]$$

The fuel consumed at time t, F_t was determined with Eq. S.31 where $F_{t-\Delta t}$ was the previously consumed fuel at time $t - \Delta t$ and flow rate consumed, $Cons^F$ (Eq. 8). The generation of CO_2 due to the combustion of the fuel was obtained with Eq. S.32 according to the CO_2 generated per ml of fuel, $UGen_{CO_2}$. A mass balance of CO_2 was also carried out to estimate the total CO_2 generated, as can be seen on Eq. S.33 were CO_2^t and $CO_2^{t-\Delta t}$ are the moles of carbon dioxide at time t and $t - \Delta t$; Q_{CO_2} the flow rate of carbon dioxide; $mol_{CO_2}^{out}$, the output moles of CO_2 at time t; and the rest of parameters previously explained. CO_2^t and $CO_2^{t-\Delta t}$, $OH^{-\,t}$ and $OH^{-\,t-\Delta t}$, $HCO_3^{-\,t}$ and $HCO_3^{-\,t-\Delta t}$ represented the moles of CO_2, OH- and carbonate/hydrogen carbonate (since both of then can be formed depending on the CO_2 to OH- ratio) at time t and $t - \Delta t$ respectively. The parameter m represented the stoichiometric factor for the reaction between CO_2 and OH^-

.

$$F^t = F^{t-\Delta t} + \Delta t \cdot \frac{Cons_F}{3600} \quad [Eq.\ S.31]$$

$$Gen_{CO_2}^t = F^t \cdot UGen_{CO_2} \quad [Eq.\ S.32]$$

$$CO_2^t = CO_2^{t-\Delta t} + \Delta t \cdot \left(\frac{Q_{CO_2} \cdot p}{R \cdot T} + \frac{Cons_F \cdot UGen_{CO_2}}{44 \cdot 3600} - mol_{CO_2}^{out} \right) \quad [Eq.S.33]$$

$$CO_2^t = CO_2^{t-\Delta t} - \frac{OH^{-\,t}}{m} \quad [Eq.\ S.34]$$

$$HCO_3^{-\,t} = HCO_3^{-\,t-\Delta t} + \frac{OH^{-\,t}}{m} \quad [Eq.S.35]$$

$$OH^{-\,t} = OH^{-\,t-\Delta t} - CO_2^t \cdot m \quad [Eq.\ S.36]$$

$$HCO_3^{-\,t} = HCO_3^{-\,t-\Delta t} + CO_2^t \cdot m \quad [Eq.\ S.37]$$

Error calculation

$$TSS = \sum \left(y_{exp}^i - \overline{y_{exp}}\right)^2 \quad [Eq.\ S.38]$$

$$RSS = \sum \left(y_{exp}^i - y_{mod}^i\right)^2 \quad [Eq.\ S.39]$$

$$R^2 = 1 - \frac{RSS}{TSS} \quad [Eq.\ S.40]$$

$$MAE = \frac{1}{n}\sum_{i=1}^{n} \left|y_{exp}^i - y_{mod}^i\right| \quad [Eq.\ S.41]$$

$$RMSE = \sqrt{\frac{1}{n}\sum_{i=1}^{n} \left(y_{exp}^i - y_{mod}^i\right)^2} \quad [Eq.\ S.42]$$

Where y_{exp}^i and y_{mod}^i represented each experimental and modeled value and $\overline{y_{exp}}$ the average of all experimental values and n the number of experimental values. TSS and RSS corresponded to the total sum of squares and the residual sum of squares, respectively.

$$RMSRE = \sqrt{\frac{1}{n}\sum_{i=1}^{n} \left(\frac{y_i^x - y_i^{30}}{y_i^{30}}\right)^2} \quad [Eq.\ S.43]$$

$RMSRE$ represented the root mean square relative error, y_i^x corresponded to the value obtained with the model with $\Delta t = x\ s$ and y_i^{30} to the value obtained with the model with $\Delta t = 30\ s$ which was set as reference.

S2. Figures

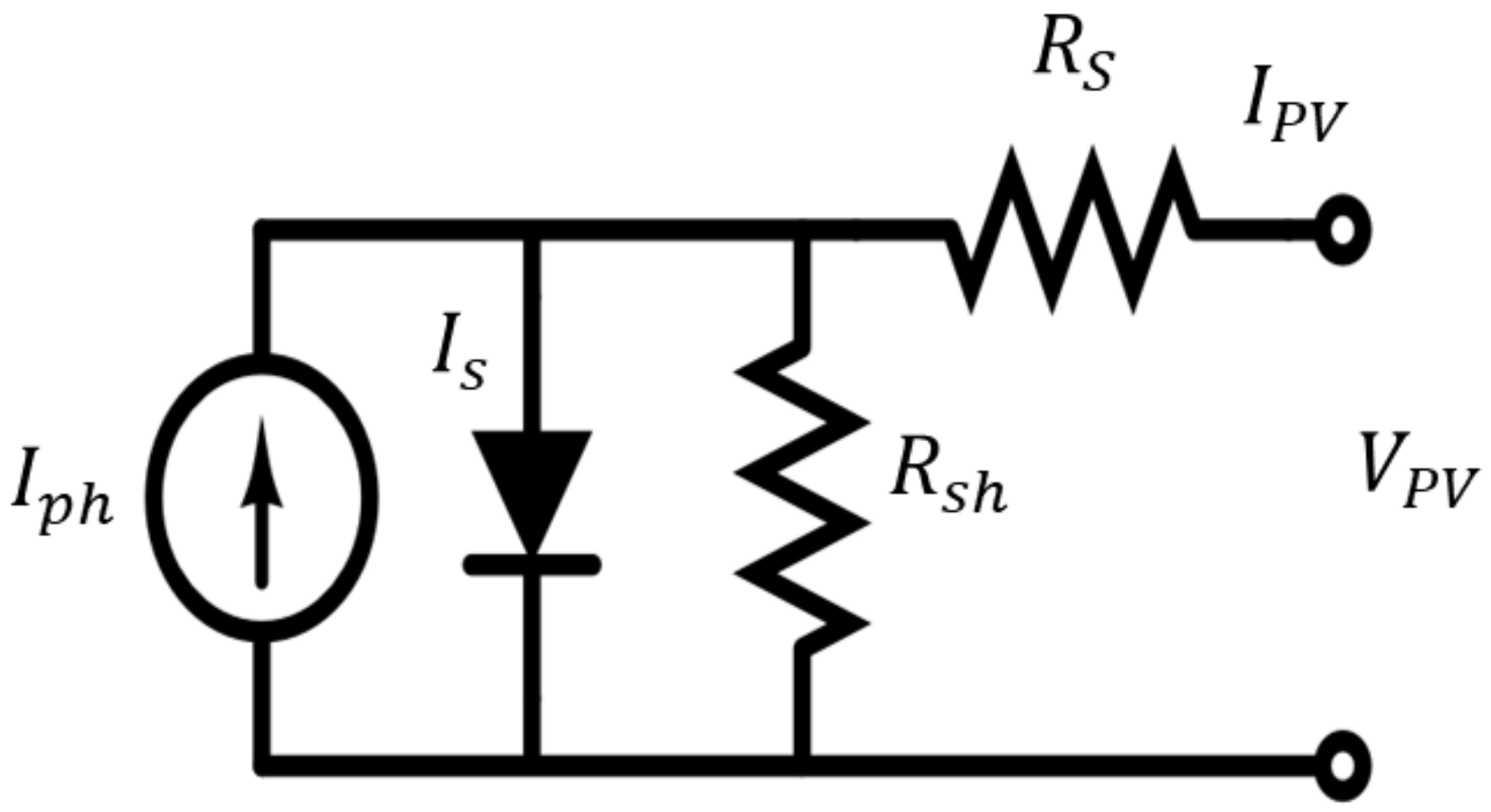

Figure S.1. Equivalent circuit for a photovoltaic panel, were I_{ph} correspond to the current in the photodiode, I_s to the saturation current, R_{sh} to the shunt resistance, R_s to the resistance in series and I_{PV} and V_{PV} to the current and voltage of the photovoltaic panel, respectively.

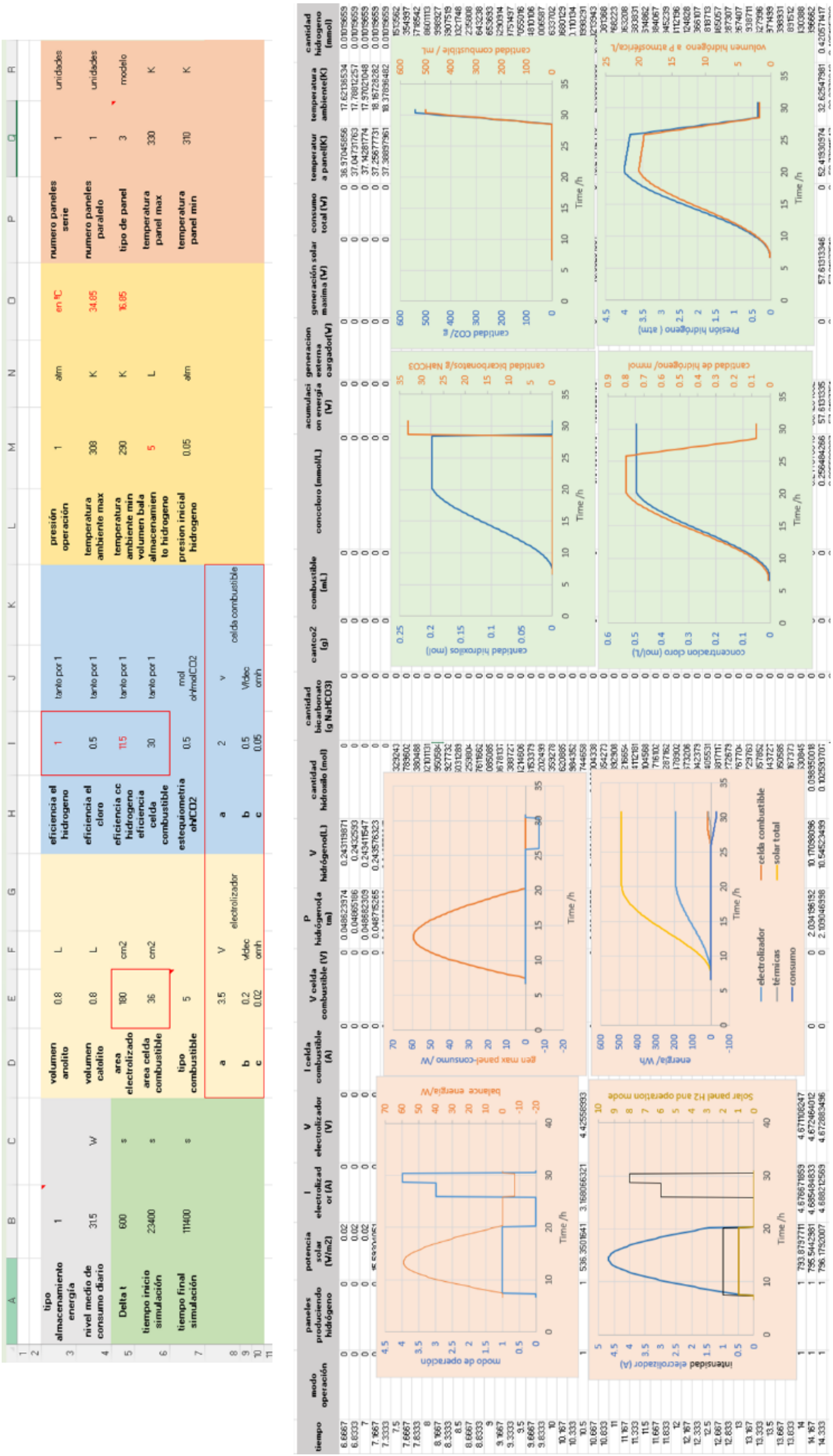

Figure S.2. Illustrative screenshots of the software developed in Excel VBA. Case study 1.

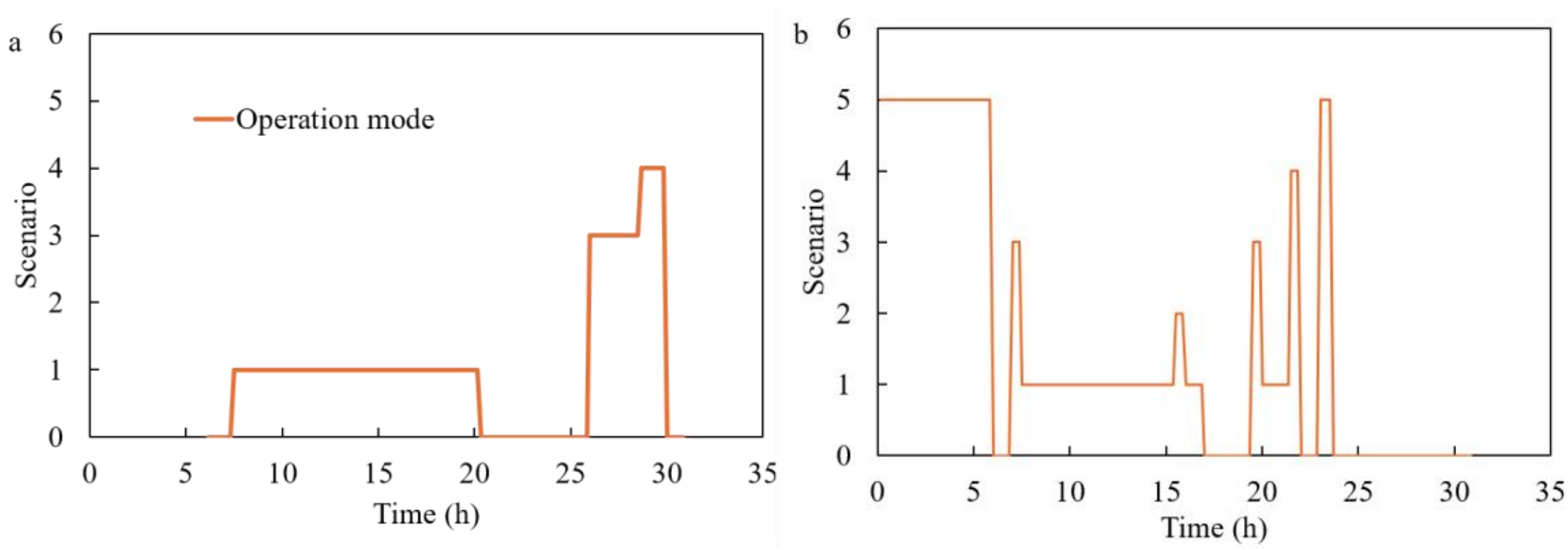

Figure S.3. Operation mode and number for all the experiment simulated with the model according to the availability of solar power and H_2 storage. To know the operational scenarios consult Table 1 a) Case study 1 and b) Case study 2.

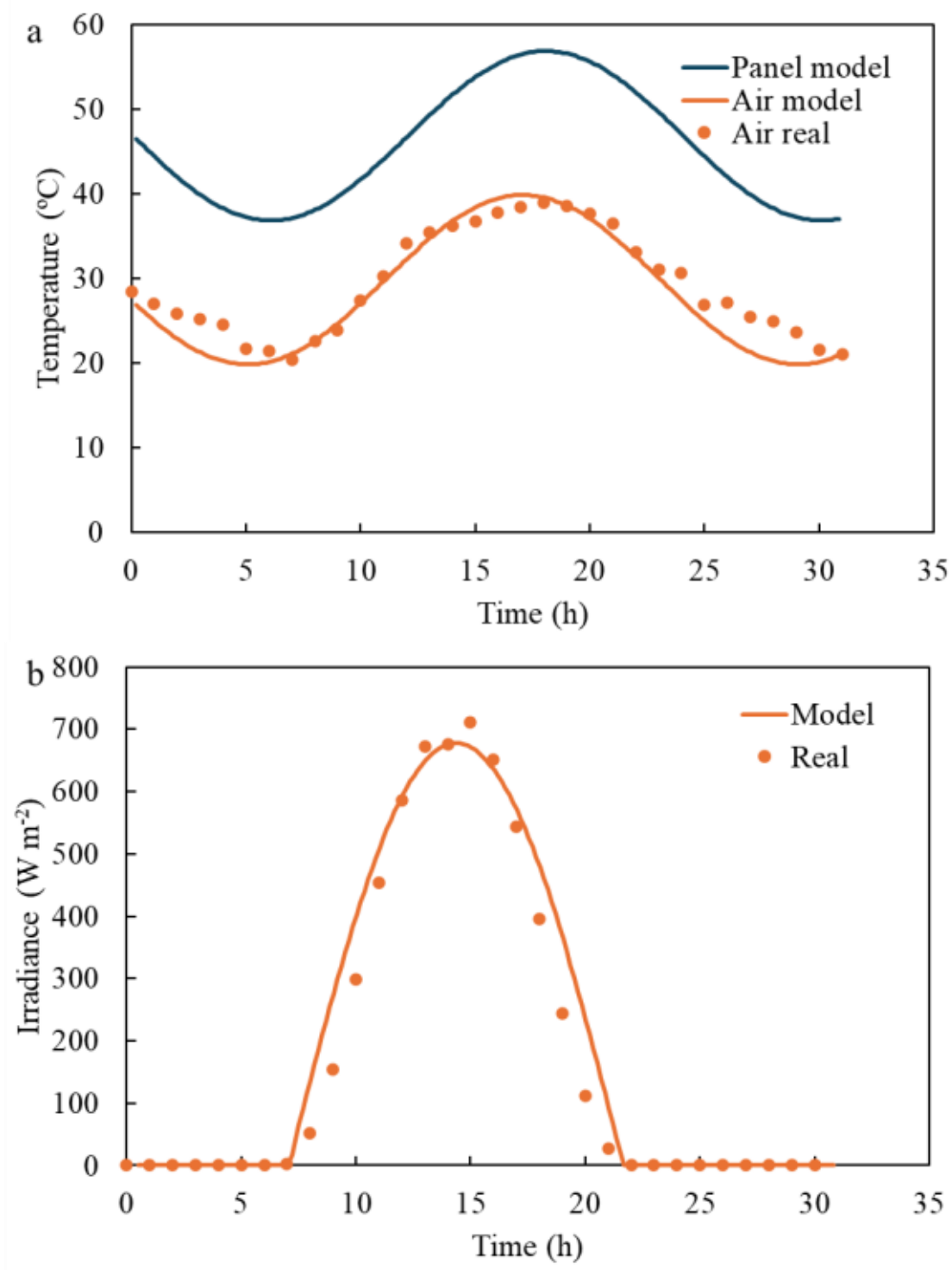

Figure S.4 a) Continuous line: temperature of air predicted with Eq. 2 and on the surface of the panel, predicted with Eq. S.3; and dash line real temperature of air; b) continuous line: Solar irradiance predicted with Eq. 1; and dash line: real irradiance for the day 15th of June 2025 for the case study two.

S3. Tables

Table S.1. Technical data for different solar panels used in the model.

Description (Units)	Variable	10 W	50 W	100 W	200 W	400 W
**Resistance (Ω)**	R_s	0.379	0.379	0.379	0.379	0.379
**Shunt Resistance (Ω)**	R_{sh}	1000	1000	1000	1000	1000
**Nº photovoltaic cells**	N_c	36	36	72	72	120
**Ref. solar power (W m^{-2})**	λ_{ref}	1000	1000	1000	1000	1000
**Reverse saturation current (A)**	I_{rs}	$3\cdot10^{-4}$	$3\cdot10^{-4}$	$3\cdot10^{-4}$	$3\cdot10^{-4}$	$3\cdot10^{-4}$
**Short circuit current (A)**	I_{SC}	0.6	2.96	5.94	7.5	9.6
**Open circuit potencial (V)**	V_{OC}	0.7	0.7	0.7	0.7	0.7
**Ideality factor**	n	1.7	1.7	1.7	1.7	1.7
**Short circuit coefficient**	K_I	$1\cdot10^{-4}$	$1\cdot10^{-4}$	$1\cdot10^{-4}$	$1\cdot10^{-4}$	$1\cdot10^{-4}$
**Current to start iteration (A)**	I_{PV}	0.56	2.78	5.56	6.8	10.94
**Voltage for max power (V)**	V_{PV}	12.1	12.1	18.01	30	34.3
